# *In Silico* Analysis of Serum Albumin Binding by Bone-Regenerative Hyaluronan-Based Molecules

**DOI:** 10.3390/pharmaceutics17111445

**Published:** 2025-11-08

**Authors:** Pauline Kramp, Aydin Özmaldar, Gloria Ruiz-Gómez, M. Teresa Pisabarro

**Affiliations:** Structural Bioinformatics, BIOTEC TU Dresden, Tatzberg 47/51, 01307 Dresden, Germany; pauline.kramp@tu-dresden.de (P.K.); aydin.oezmaldar@tu-dresden.de (A.Ö.)

**Keywords:** bone-regenerative GAG, _RE_GAG, chemically modified hyaluronan, serum albumin binding, molecular docking, molecular dynamics simulation

## Abstract

**Background:** The binding of glycosaminoglycans (GAG) to Wnt signaling components plays a key regulatory role in bone formation and regeneration. We previously reported *de novo* designed chemically modified hyaluronan derivatives, named _RE_GAG (Rationally Engineered GAG), which demonstrated bone-regenerative properties in a mouse calvaria defect model. To gain initial insights into the pharmacological profile of two _RE_GAG currently under preclinical investigation in mice, we performed a comprehensive *in silico* investigation of their binding to human and murine serum albumin (HSA and MSA), as it might influence their ADME properties. Furthermore, we evaluated whether _RE_GAG binding might impact the recognition of well-characterized HSA-binding drugs. **Methods**: State-of-the-art *in silico* ADMET tools, docking and molecular dynamics simulations were used to predict and characterize the interaction of _RE_GAG with HSA and MSA, and to investigate the molecular mechanisms involved at the atomic level. **Results**: The investigated _RE_GAG molecules show a consistent binding preference for the FA1 site in both proteins, and an additional preference for the FA7 site in HSA. Their recognition might induce protein conformational changes and alter the functional state. Furthermore, _RE_GAG’s conformational adaptability is predicted to influence their binding to the FA5/6 and FA8/9 sites of HSA, and to the FA3/4 and FA7 sites of MSA. **Conclusions**: Our investigations predict the binding of two hyaluronan derivatives to HSA and MSA. The mechanistic insights gained into the molecular recognition of these two _RE_GAG molecules offer valuable information for their potential clinical application and serve as a rational basis for future molecular design aimed at improving pharmacokinetic properties.

## 1. Introduction

**Figure 1:**
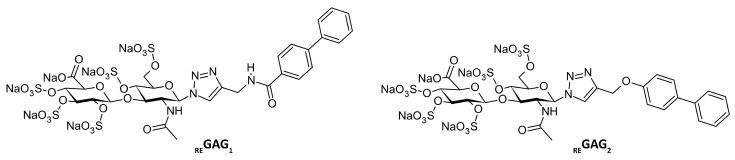
Chemical structure of the bone-regenerative _RE_GAG variants _RE_GAG_1_ and _RE_GAG_2_.

Rationally engineered glycosaminoglycan derivatives (_RE_GAG) have been recently reported as promising molecules for bone regeneration by targeting key inhibitors of the Wnt/β catenin signaling pathway. *In vitro* and *in vivo* studies showed that two of these _RE_GAG variants, named _RE_GAG_1_ and _RE_GAG_2_ (Figure 1), outperform synthetic polymeric highly sulfated hyaluronan by achieving up to 50% bone regeneration in a mouse calvaria defect model [1]. These _RE_GAG molecules can restore Wnt signaling by interacting with and inhibiting the Wnt negative regulators Dickkopf-1 (DKK-1) and sclerostin [1], which function cooperatively and compete with activating Wnt ligands for binding sites on the receptors low-density lipoprotein receptor related protein 5 and 6 (LRP5/6) [2,3,4,5]. Of note, a dual inhibition of both Wnt negative regulators is crucial to enhance Wnt signaling and promote synergistic bone formation, compared to monotherapies targeting either DKK-1 or sclerostin separately [6,7]. _RE_GAG mimic the LRP5/6 receptor region recognized by DKK-1 *C*-term and several Wnt activating ligands. Because of several recognition resemblances to LRP5/6 regions used by DKK-1 and sclerostin, _RE_GAG are able to scavenge both endogenous inhibitors of the Wnt/β catenin signaling pathway, thereby preventing its inhibition and promoting osteogenesis [1].

The chemical structure of _RE_GAG_1_ and _RE_GAG_2_ consists of a sugar-based moiety, a pentasulfated hyaluronan disaccharide functionalized at the anomeric center, with either 1,2,3-triazol-amide or ether linkers connecting it to a hydrophobic aromatic moiety (Figure 1).

Given the therapeutic potential of _RE_GAG_1_ and _RE_GAG_2_ in bone-related diseases such as osteoporosis [8], further investigation into their pharmacological properties is warranted. A key step in early drug development involves determining ADMET (absorption, distribution, metabolism, excretion, and toxicity) properties to evaluate the safety and efficacy of novel drug candidates. In this regard, computational methods can be used to predict several ADMET properties and thus reduce experimental work costs [9]. A key parameter in computational ADMET profiling is the determination of plasma protein binding. In this context, human serum albumin (HSA) constitutes a key target as it plays a central role in drug transport, distribution, and clearance. Furthermore, HSA is the most abundant plasma protein (500–700 µM), capable of binding a wide range of endogenous and exogenous ligands, including fatty acids and drugs [10,11,12]. Therefore, the *in silico* investigation of the interaction of _RE_GAG_1_ and _RE_GAG_2_ (Figure 1) with HSA represents an important step toward advancing understanding of their pharmacological profiles. Furthermore, because ligand-binding characteristics can differ between human and mouse serum albumins, the successful translation of preclinical findings from mouse models to human applications requires a rigorous comparison of ligand recognition in both species. State-of-the-art computational strategies provide an efficient and cost-effective first step for examining ligand recognition by both the human and mouse serum albumin proteins and identifying potential species-specific interactions. Such insights are valuable for prioritizing candidates, guiding experimental validation, as well as supporting future optimization through the lead-to-drug development process.

**Figure 2:**
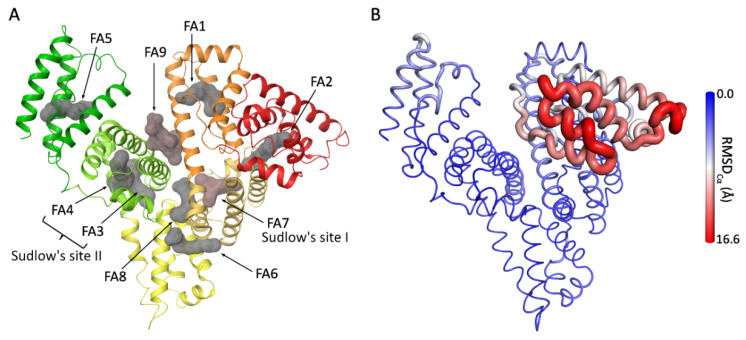
HSA structural features. (**A**) Crystallographic structure of HSA in complex with both endogenous and exogenous ligands at the fatty acids binding sites (PDB ID 1H9Z, 2.5 Å). HSA is shown in cartoon (subdomain IA in red, IB in orange, IIA in faded orange, IIB in yellow, IIIA in green-yellow, and IIIB in green). Ligands at the different binding sites (indicated by arrows and labeled) are shown as molecular surfaces (in gray: myristic acid at FA1- FA6 and Decanoic acid at FA8 (taken from PDB ID 1E7E, 2.5 Å); in red-brown: warfarin at FA7 and thyroxine at FA9 (taken from PDB ID 1HK4, 2.4 Å)). Figure generated with Maestro v14.3. (**B**) Cartoon putty representation of HSA colored and rendered according to RMSD_Cα_ values (indicated by the gradient side bar) relative to the comparison between its complex with propofol at subdomain IIIA (PDB ID 1E7A, 2.2 Å) and with heme-Fe(III) at subdomain IB (PDB ID 1N5U, 1.9 Å). Figure generated with PyMOL v2.3.

The 3D structure of murine serum albumin (MSA) has not been yet experimentally solved. However, there are several high-resolution crystallographic structures of its human homologue, HSA, available in the Protein Databank. HSA consists of six different subdomains (IA (residues 5–107), IB (residues 108–197), IIA (residues 198–296), IIB (residues 297–382), IIIA (residues 383–494), and IIIB (residues 495–569) [13]), which are connected by flexible loops (Figure 2). HSA’s well-characterized structure features three main drug-binding sites (Sudlow sites I and II in subdomains IIA and IIIA, respectively, as well as drug site III in subdomain IB) and nine fatty acid (FA) binding pockets (FA1–FA9), some of which are located at the subdomain interfaces (Figure 2A) [14,15,16,17]. Notably, HSA is an allosterically regulated protein (*i.e.*, ligand binding at one site can alter the binding properties at others). For example, binding of ferric heme at FA1 significantly reduces drug affinity at Sudlow’s site I and, conversely, drugs binding at Sudlow’s site I can hinder ferric heme association [10]. Fatty acids also modulate ligand binding, as Sudlow site I ligands preferentially bind the FA-free state, while FA1 ligands show enhanced binding in the FA-bound conformation [10,18].

Due to its allosteric nature, HSA can undergo conformational changes in response to variations in pH and ligand binding (Figure 2B) [19]. Indeed, two well-defined conformational states have been characterized. The “neutral” (N) form occurs at pH 7 in the FA-free state, and the “basic” (B) form appears at pH higher than 8 in the FA-free state or at neutral pH in the FA-bound state [20]. In addition, occupation of key HSA binding sites can significantly alter binding behavior, thereby affecting the distribution of other ligands and modulating HSA capacity to transport metabolites and drugs [15,21,22,23]. HSA’s conformational diversity is extensively represented in the structures available at the Protein Data Bank (PDB), revealing major structural rearrangements within subdomains I and III [15,16,24,25,26,27,28], particularly in their orientation with respect to subdomain II, which acts as a structural pivot during conformational transitions [20,29]. Fatty acid binding to the FA2 site between the interface of subdomains IA and IIA has been considered one of the major forces of this conformational change [27].

Here, we investigate the pharmacological properties of _RE_GAG_1_ and _RE_GAG_2_ *in silico* by characterizing their ability to interact with HSA in different conformational states and assessing their potential to alter the binding of clinically relevant reference drugs. Furthermore, we analyze the interactions of these _RE_GAG molecules with murine serum albumin (MSA), a critical aspect for ongoing preclinical studies and for supporting potential future translation to human applications.

## 2. Materials and Methods

### 2.1. In Silico Prediction of _RE_GAG Binding to Plasma Proteins

The web-based platforms ADMET-AI [30], ADMETlab [31], Deep-PK [32], and admetSAR [33] were employed to predict plasma protein binding for the two investigated _RE_GAG molecules. These tools make use of classical computational approaches as well as machine learning and deep learning algorithms, as implemented in ADMET-AI and Deep-PK.

### 2.2. Molecular Modeling of Murine Serum Albumin (MSA)

The three-dimensional (3D) structure of MSA was built by comparative modeling using as template the crystallographic structure of HSA in complex with aristolochic acid (PDB ID 8RCO, 1.9 Å) [25] (72% sequence identity and 89% similarity). The program MODELLER as implemented in BIOVIA [34] was used for the modeling. The obtained MSA model was compared to the MSA structure predicted by AlphaFold (AF-P02768-F1-v4) [35,36].

### 2.3. Molecular Docking

Given that HSA is allosterically regulated and undergoes ligand-induced conformational changes [18], the ligand-bound structures available at high resolution in the PDB offer a variety of conformational variations in the respective subdomains. Therefore, we selected three representative crystallographic structures of HSA that capture distinct initial conformational states relevant to particular ligand-binding subdomains and reflect conformational variability between domains I and III. The warfarin- and myristic acid-bound HSA structure (PDB ID 1H9Z, 2.5 Å) [27] was chosen because it reflects the ligand-bound conformational state across FA1-FA7. A second structure comprising heme-Fe(III) bound in FA1 and myristic acid occupying FA2-FA6 (PDB ID 1N5U, 1.9 Å) [28] was also included because it represents a ligand-free conformation of drug binding site I (FA7) within subdomain IIA, while maintaining ligand-bound conformations in adjacent subdomains. Finaly, the propofol-bound structure (PDB ID 1E7A, 2.2 Å) [24] was selected because it represents the conformational state with ligands exclusively occupying the FA3/4 (drug-binding site II) and FA5 sites. Furthermore, these crystal structures have been employed in previous molecular docking and dynamics studies of HSA–ligand complex [37,38,39], thereby supporting their suitability for comparative docking in the present work. In the selected structures, the co-crystallized ligands were removed prior to protein preparation. Additionally, the fatty acid- and heme-Fe(III)–containing HSA structure (PDB 1H9Z) was selected for molecular docking to investigate the molecular recognition of the _RE_GAG molecules in a ligand-bound conformational state. All structures were prepared in BIOVIA [34] and refined in QuickPrep from MOE [40] using the Amber14 forcefield [41], the Generalized Born implicit solvation model [42] and additional default parameters.

Given the hybrid structure of the _RE_GAG_1_ and _RE_GAG_2_ molecules, which include both a GAG and a non-GAG moiety, three different but complementary docking approaches were employed: Glycotorch Vina [43], a specialized tool for investigating GAG–protein interactions, and Autodock 3 [44] and Glide [45,46], which are optimized for small-molecule docking. The combined use of these three methods ensured a more complete and reliable representation of the binding properties of the investigated _RE_GAG molecules. The structures of _RE_GAG_1_ and _RE_GAG_2_ were prepared for Glycotorch using AutoDock Tools [47]. In Glide, their 3D structures were prepared in LigPrep from the Maestro suite (v14.3.129, Release 2025-1) [48] using the OPLS4 force field [49] and Epik [50] to generate ionization states at pH 7.4 ± 2.0. For comparison and validation purposes, heme-Fe(III), cantharidin, warfarin, propofol and flurbiprofen were also prepared in LigPrep.

The ligands were treated flexibly. In Glycotorch and Autodock 3, the proteins were treated rigidly, while in Glide flexible rotation of hydroxyl and thiol groups of Ser, Thr, Tyr and Cys was allowed. In GlycoTorch, the size box dimensions for the ligand-free HSA structures PDB IDs 1H9Z, 1N5U, and 1E7A were set up to 50.0 Å × 50.0 Å × 50.0 Å, 48.6 Å × 48.6 Å × 48.6 Å, and 49.4 Å × 49.4 Å × 49.4 Å, respectively. For warfarin- and myristic acid-bound HSA (PDB ID 1H9Z), the size box dimension was set up to 84.3 Å × 84.3 Å × 84.3 Å. For MSA, a size box dimension of 63.4 Å × 63.4 Å × 63.4 Å was used. The exhaustiveness of the search algorithm and number of decoys were set up to 100. For Autodock 3 calculations, autogrid3 was used to compute the atomic potential of HSA PDB ID 1H9Z with a spacing grid and grid box of 0.397 Å and 126 Å × 126 Å × 126 Å, respectively. Glide docking calculations were carried out in standard precision. The grid boxes were centered (*x*: 28.578, *y*: 12.695, *z*: 7.549 for PDB ID 1H9Z; *x*: 26.783, *y*: 3.931, *z*: 20.932 for PDB ID 1N5U; *x*: 1.166 *y*: 11.600, *z*: −12.133 for PDB ID 1E7A; *x*: 28.782, *y*: 2.499, *z*: 7.995 for the MSA model centering FA1; *x*: 13.008, *y*: 4.881, *z*: 10.543 for the MSA model centering FA7, and *x*: 41.519, *y*: −0.940, *z*: 25.809 for the MSA model centering subdomain IIIA) with inner and outer boxes of dimensions 25.0 Å × 25.0 Å × 25.0 Å and 53.0 Å × 53.0 Å × 53.0 Å, respectively, for PDB ID 1H9Z, 29.0 Å × 29.0 Å × 29.0 Å and 65.0 Å × 65.0 Å × 65.0 Å, respectively, for PDB ID 1E7A, and 40.0 Å × 40.0 Å × 40.0 Å and 76.0 Å × 76.0 Å × 76.0 Å, respectively, for PDB ID 1N5U and the MSA model. In Glycotorch and Autodock 3, the top 50 docking solutions were clustered with DBSCAN as previously described [51]. In Glide, the top 20 best docking solutions were kept and analyzed. Three binding poses from each cluster were selected as representative _RE_GAG/protein complex structures for further MD refinement. Docking results figures were created with Maestro v14 [48].

### 2.4. Molecular Dynamics (MD) Simulations

The selected representative _RE_GAG/HSA and _RE_GAG/MSA complex structures were energetically refined by MD simulations in AMBER19 [52]. Charges were taken from the GLYCAM 06-j force field [53], and from the literature for sulfate groups [54]. Parameters for the GAG moiety were taken from the GLYCAM-06j force field [53], and protein parameters from the ff14SB force field [41]. Parameters for the GAG-anomeric functionalities were taken from previous work [1]. Each _RE_GAG/protein complex was solvated in a truncated octahedral box of TIP3P water molecules and neutralized with Na^+^ counterions. MD simulations were preceded by two energy-minimization steps: (*i*) only the solvent and ions were relaxed with position restraints for the solute (500 kcal/mol·Å^2^) using 1000 steps of steepest descent minimization followed by 500 steps of conjugate gradient minimization; (*ii*) the entire system was minimized without restraints applying 3000 cycles of steepest descent and 3000 of conjugate gradient equilibration. Then, the system was heated up from 200 K to 300 K in 20 ps with weak position restraints (10 kcal/mol·Å^2^). Langevin temperature coupling with a collision frequency γ = 1 ps^−1^ was used at this step. The system was equilibrated under constant pressure of 1 atm using periodic boundary conditions (NPT conditions) at 300 K for 500 ps. A total of 100 ns MD simulation was carried out at 300 K with NPT conditions for each complex. The SHAKE algorithm was used to constrain all bonds involving hydrogen atoms with a time step of 2 fs. A cutoff of 8 Å was applied to treat the non-bonded interactions, and the Particle Mesh Ewald (PME) method was used to treat long-range electrostatic interactions. MD trajectories were recorded every 10 ps. The pyranose rings in the _RE_GAG molecules were harmonically restrained. Trajectories were visualized with VMD [55] and evaluated in terms of RMSD and principal component analysis using the CPPTRAJ module implemented in AMBER19 [52]. Pairwise energy decomposition and binding free energy post-processing analysis of 200 frames distributed along the MD production runs were performed in implicit solvent using MM-GBSA [56,57] as implemented in AMBER19. The Pandas, NumPy, and Matplotlib packages were utilized as tools for data manipulation, analysis, and visualization. Figures were created with PyMOL v2.3 [58].

## 3. Results

### 3.1. In Silico Predictions of _RE_GAG Binding to Plasma Proteins

_RE_GAG_1_ and _RE_GAG_2_ plasma protein binding was investigated using several web-based ADMET platforms (see Methods Section 2.1, Table 1). Since these molecules are *de novo* designed, it should be noticed that the predictions made rely entirely on the models and training sets used by the respective tools and may be biased depending on the chemical features represented in those datasets.

The probability of _RE_GAG_1_ and _RE_GAG_2_ binding to plasma proteins was predicted medium to high. The obtained results indicated greater variability for _RE_GAG_1_ in comparison to _RE_GAG_2_, suggesting moderate and good predictive reliability for _RE_GAG_1_ and _RE_GAG_2_, respectively [59].

### 3.2. Molecular Recognition of _RE_GAG by HSA

Docking calculations were carried out to investigate in detail the molecular recognition of _RE_GAG_1_ and _RE_GAG_2_ by HSA. For this purpose, considering that the _RE_GAG molecules contain GAG and non-GAG moieties, we used different docking programs that have been designed for predicting binding of GAGs (*i.e.*, GlycoTorch) [43] and/or small molecules (*i.e.*, Glide, Autodock 3) [44,45,60]. Moreover, the predictive capacity of Glide was further assessed by blind docking calculations with five known HSA-binding ligands (see Methods Section 2.3, Figure S1), confirming its robustness in identifying previously reported HSA recognition sites. The representative _RE_GAG/HSA complexes obtained were further optimized by MD simulations (see Methods Section 2.4) to evaluate their stability and characterize the strength of the interactions between the _RE_GAG molecules and HSA at the predicted recognition sites. In particular, we investigated the interactions of _RE_GAG_1_ and _RE_GAG_2_ with HSA at the following binding sites: drug site I (FA7), FA6 and FA8 in subdomain II (using the HSA structure from PDB ID 1H9Z and 1N5U, which differ in their co-crystallized ligands and occupied FA sites, see Methods Section 2.3), drug site II (FA3/4) and FA5 binding site in subdomain III, as well as FA9 between subdomains I and III (using PDB ID 1E7A), and the drug site III (heme site/FA1) in subdomain I (using PDB ID 1N5U and 1H9Z) and FA2 site between subdomains I and IIA (using PDB ID 1N5U). Structural superimposition and comparative analysis of the selected HSA crystallographic structures revealed that the largest conformational changes occur in subdomain I, which exhibited the highest RMSD_Cα_ values (Table S1 and Figure 2B).

Docking results suggested that _RE_GAG_1_ and _RE_GAG_2_ would not compete with ligands that recognize subdomain IA, the FA2 site and drug-binding site II (Figure 2A), as no binding poses were observed in those regions. The obtained docking poses for the _RE_GAG molecules clustered at drug-binding site III (heme site), drug-binding site I (Sudlow’s site I), and fatty acid binding sites FA5, FA6, FA8, and FA9 in different extents. Detailed results are provided in the following subsections:

#### 3.2.1. Subdomain IB (Drug Site III/Heme/FA1 Site)

In the absence of the native heme ligand at HSA subdomain IB (PDB ID 1N5U), docking of _RE_GAG_1_ and _RE_GAG_2_ with GlycoTorch suggested that the biphenyl groups of the _RE_GAG molecules could act as anchoring moieties through π–π interactions with residues Tyr138 and Tyr161 of HSA, which are known to be key for heme recognition [28] (Figure S2). Interestingly, docking calculations with Glide only predicted binding poses at that site for _RE_GAG_2_. Furthermore, the Glide docking score obtained for the interaction of heme with HSA (−12.2 ± 1.8 kcal/mol) was more favorable than for _RE_GAG_2_ (−5.5 ± 3.4 kcal/mol) and the reported monoterpene cantharidin competitive ligand [61] (−5.3 ± 0.3 kcal/mol). These results suggest that, in ligand-free HSA, the _RE_GAG molecules might compete for the heme site but are unlikely to displace the bound heme-Fe(III).

**Figure 3:**
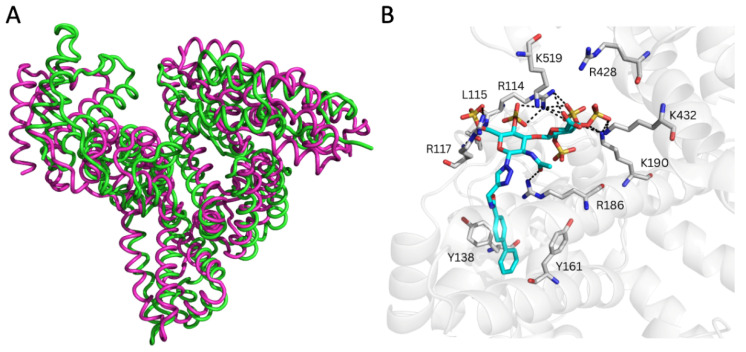
Molecular recognition of _RE_GAG_1_ by HSA (PDB ID 1N5U) at the heme (FA1) site. (**A**) Superimposition of two MD-refined HSA structures (green and pink ribbons) highlighting conformational displacements relating to the first five principal components obtained from principal component analysis over 100 ns MD simulations of _RE_GAG_1_ in complex with HSA. (**B**) Representative MD-refined structure of _RE_GAG_1_ (in cyan sticks colored by atom type) in complex with HSA (transparent gray cartoon). Relevant interacting HSA residues are shown in gray sticks and colored by atom type. H-bonds are depicted with dashed black lines. Figure generated with PyMOL v2.3.

**Figure 4:**
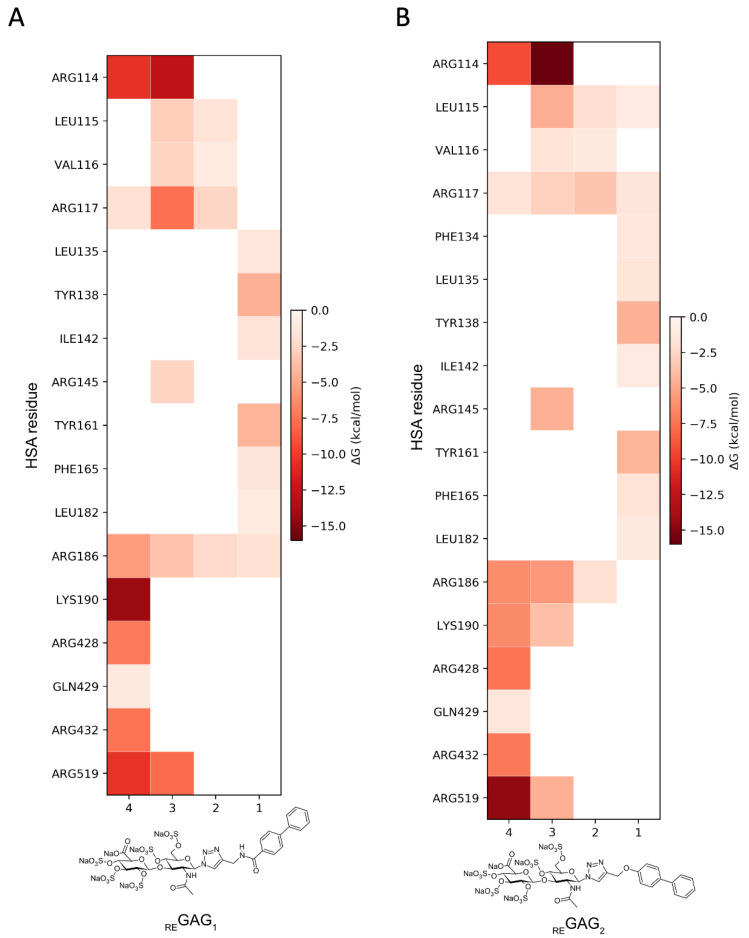
Pairwise binding free energy contributions calculated with MM-GBSA from three independent 100 ns MD simulations of HSA (PDB ID 1N5U) in complex with (**A**) _RE_GAG_1_ and (**B**) _RE_GAG_2_ bound to the heme site (FA1). Mean values are indicated by the gradient-colored side bar. The different fragments of each _RE_GAG molecule are represented in the *x* axis by the numbers: (1) biphenyl group, (2) linker, (3) fully sulfated N-acetylglucosamine (GlcNAc), and (4) fully sulfated glucuronic acid (GlcA).

Moreover, the analysis of the representative _RE_GAG/HSA complexes predicted by GlycoTorch and refined by MD revealed large structural fluctuations after approximately 50 ns of simulation, especially in subdomain IIIB as clearly inferred from the principal component analysis (Figure 3, Figures S3 and S4). This indicates that this region contributes most strongly to the dominant collective motions captured in the simulation and, therefore, it reflects possible rearrangements of the protein–ligand complexes over time. The free energies of binding along the MD trajectories computed with MM-GBSA [56,57] for the _RE_GAG molecules (see Methods Section 2.4) indicated comparable binding (*i.e.*, ΔG_RE_GAG_1_ = −52.8 ± 1.6 kcal/mol; ΔG_RE_GAG_2_ = −56.6 ± 4.6 kcal/mol; Table 2). The binding of each _RE_GAG molecule to the heme site involved HSA residues across subdomains I and III, reflecting a cooperative interdomain interaction network. In particular, the disaccharide moiety of both _RE_GAG molecules interacted with HSA residues Arg114, Leu115, Arg117, Arg145, Arg186, Lys190, Arg428, Lys432, and Lys519, while the biphenyl groups formed a stable sandwich between Tyr138 and Tyr161 (Figure 3B and Figure 4).

#### 3.2.2. Subdomain IIA (Drug-Binding Site I/Warfarin/FA7 Site)

GlycoTorch predicted a main cluster for _RE_GAG_1_ and _RE_GAG_2_ binding at the drug-binding site I of HSA (PDB ID 1H9Z), whereas no binding poses were identified at the FA7 site when using Glide. This, instead, predicted binding poses surrounding the FA7 pocket (Figure S5). To gain further insights into these differing predictive results, we performed additional studies using Autodock 3 (Figure S6), whose results resembled those from Glide.

**Figure 5:**
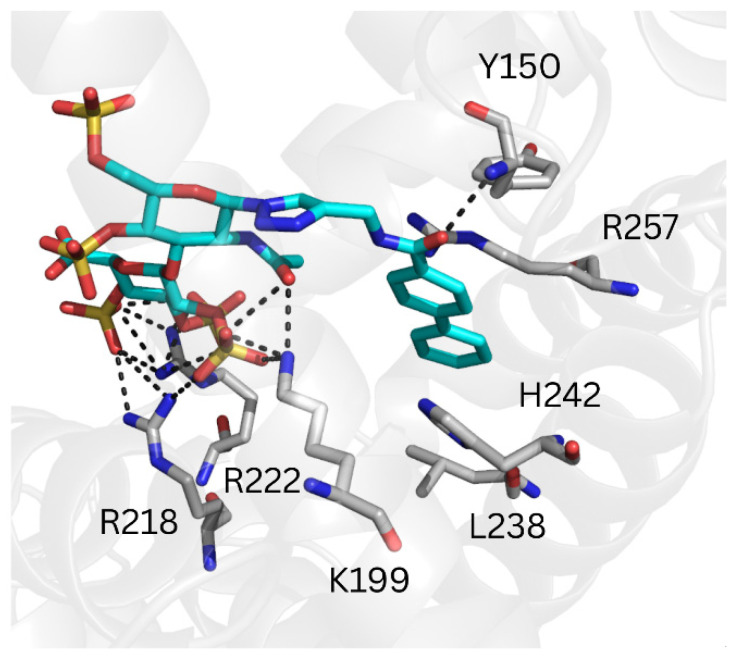
Representative MD-refined structure of _RE_GAG_1_ in complex with HSA (transparent gray cartoon) at the warfarin (FA7) site. Relevant interacting HSA residues are shown in gray sticks and colored by atom type. _RE_GAG_1_ is shown in cyan sticks and colored by atom type. H-bonds are depicted with dashed black lines. Figure generated with PyMOL v2.3.

The binding poses predicted by GlycoTorch positioned the GAG moiety of the _RE_GAG molecules at the same site, while their respective non-GAG moieties adopted different binding modes (Figure S5A). The computed free energies of binding suggested that _RE_GAG_1_ binding to HSA drug-site I was slightly more favorable than _RE_GAG_2_ (*i.e.*, ΔG_RE_GAG_1_ = −57.9 ± 10.2 kcal/mol for one recognition mode; ΔG_RE_GAG_1_ = −39.3 ± 7.3 kcal/mol for another recognition mode; ΔG_RE_GAG_2_ = −49.1 ± 9.8 kcal/mol; Table 2). Furthermore, the pairwise binding energy decomposition analysis obtained from the MD trajectories indicated that the GAG moiety of the _RE_GAG molecules interacts strongly with the basic residues Lys195, Lys199, Arg218, Arg222 and Lys444 (Figure S7), underscoring its relevant role for _RE_GAG recognition. Likewise, and considering the most favorable recognition mode of _RE_GAG_1_, Leu219, Leu238, His242, Arg257, Leu260, Ile290 and Ala291, which forms the hydrophobic core within Sudlow’s site I (subdomain IIA), emerged as anchor points for the recognition of the biphenyl groups in both _RE_GAG molecules (Figure 5, Figure S7A). In contrast, in the less favorable recognition mode of _RE_GAG_1_, the biphenyl group was predicted to establish interactions with Tyr150, Gln196, Lys199, Arg218, Leu238, His242 and Arg257 (Figure S7B). Notably, given that the hydrophobic residues Leu219 and Leu238 act in concert with other key residues, such as Arg218, Arg222 and His242 for (R)-warfarin recognition [27], the competition of the _RE_GAG molecules for the same site as warfarin might affect the therapeutic effect of both, _RE_GAG and the anticoagulant when used simultaneously.

As an additional control, we investigated the interaction of _RE_GAG_1_ and _RE_GAG_2_ at drug-binding site I using the HSA structure PDB ID 1N5U, which has higher resolution than 1H9Z and may more accurately reflect local conformational changes induced by their distinct co-crystallized ligands. GlycoTorch predicted similar binding poses for both molecules (Figure S2). Once the complexes were MD-refined, in comparison to those obtained with HSA PDB ID 1H9Z, they exhibited a more closed conformation of HSA, particularly within subdomains IB and IIIB. In this case, both _RE_GAG molecules showed similar binding strength to HSA (ΔG_RE_GAG_1_ = −47.6 ± 1.2 kcal/mol; ΔG_RE_GAG_2_ = −47.5 ± 14.2 kcal/mol; Table 2). Strong interactions between the HSA basic residues Lys199, Arg218, Arg222, and the GAG moieties were also predicted. On the other hand, the non-GAG moieties, especially the biphenyl groups, established interactions with Tyr148, Tyr150, Gln196, Lys199, His242, Cys245, Cys246 and Arg257 (Figure S8). These results further support that _RE_GAG_1_ and _RE_GAG_2_ may bind to the FA7 site and interfere with warfarin recognition by interacting with HSA key residues.

#### 3.2.3. Subdomain II (FA6 and FA8 Sites)

Molecular docking to HSA PDB ID 1H9Z predicted the binding of _RE_GAG_1_ and _RE_GAG_2_ at the FA6 and FA8 sites.

At the FA6 site, GlycoTorch only predicted binding poses for _RE_GAG_2_. However, Glide predicted similar binding poses for both _RE_GAG molecules, although the cluster corresponding to _RE_GAG_1_ was more populated than that of _RE_GAG_2_ (Figure S5). Interestingly, Autodock 3 (Figure S6) suggested similar binding poses to those predicted by GlycoTorch (Figure S5A). The binding poses predicted by Glide (Figure S5B) were MD-refined and further analyzed to obtain additional insights into the potential recognition of both _RE_GAG molecules at the FA6 site. The calculated free energy of binding for _RE_GAG_1_ was particularly weaker than that of _RE_GAG_2_ (ΔG_RE_GAG_1_ = −23.2 ± 1.9 kcal/mol, ΔG_RE_GAG_2_ = −38.4 ± 8.9 kcal/mol, Table 2) and also in comparison to the values obtained at the drug-binding site I, suggesting a clear lower preference of _RE_GAG_1_ for the FA6 site. For both _RE_GAG molecules, the GAG moiety established electrostatic interactions with the basic residues Arg209, Lys212, Lys233, Lys240 and Lys323. In addition, the GAG moiety of _RE_GAG_2_ interacted with Lys351, Ser480 and Asn483 (Figure S9). These contacts were mostly transient but recurrent through the MD trajectories, highlighting the flexibility and accessibility of the FA6 site. With respect to the non-GAG moiety, the biphenyl groups of both _RE_GAG molecules were positioned within the HSA hydrophobic core formed by the aliphatic parts of the side chains of Arg209, Lys212, Ala213, Asp324, Leu327, and Gly328, with additional contributions from Leu331, Ala350 and Lys351 in the case of _RE_GAG_1_ (Figure S9).

**Figure 6:**
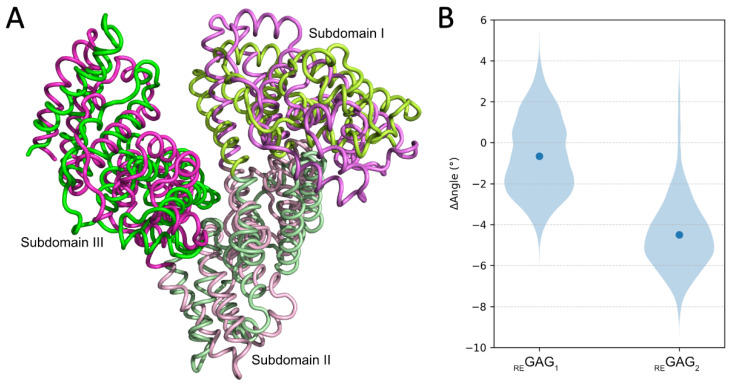
Conformational analysis of HSA in complex with _RE_GAG molecules at the FA8 site in the most favorable recognition mode. (**A**) Principal component analysis of _RE_GAG_2_ in complex with HSA. Superimposition of two MD-refined HSA structures, one shown in green and the other in pink, highlighting conformational displacements within the three subdomains—colored using gradients of the corresponding base color—captured by the first five principal components over 100 ns MD simulations. Figure generated with PyMOL v2.3. (**B**) MD-analysis of angles involving subdomains IA, IIB and IIIA of HSA in the closed conformation when complexed with the _RE_GAG molecules. Violin representation of calculated angles from 100 ns MD simulations defined by the Cα carbon of residues P110, A364 and S427 of HSA (PDB ID 1H9Z). ΔAngle values correspond to the difference between the calculated angles over the MD trajectories and the angle from the initial structure. The mean is highlighted by a blue dot.

At the FA8 site, both GlycoTorch and Glide predicted similar binding poses for _RE_GAG_2_, while _RE_GAG_1_ was predicted to bind at the neighboring FA8 site (Figure S5). In _RE_GAG_2_, the biphenyl group was predicted to interact with Trp214 via π–π stacking, whereas the GAG moiety adopted two distinct orientations, one at HSA’s surface, partially overlapping the FA6 site [17], and the other encompassing subdomains IB, II and III. The MD refinement of representative complexes suggested that _RE_GAG_2_ binds more favorably to HSA in the second recognition mode (Table 2). Furthermore, MD-based analysis confirmed the stability of the binding mode already predicted by GlycoTorch and Glide in which the biphenyl group of _RE_GAG_2_ interacts with Trp214. Additionally, interactions of the biphenyl group with residues Leu198, Lys199, Ser202 and Val344 at subdomain II, and Leu481 at subdomain IIIA (in the proximity of HSA’s C-term) were observed (Figure S10). In the binding mode with the GAG moiety at HSA’s surface, the biphenyl group also established van der Waals interactions with Lys195, Leu347 and Val482 (Figure S10B). In the binding mode with the GAG moiety located between subdomains IB, II and III, additional contacts with Phe206, Ala210 and Phe211 were predicted (Figure S10C). Of note, Phe211 has been reported as a key residue for warfarin recognition at the FA7 site [27]. In addition, in the binding mode at HSA’s surface, the GAG moieties were observed to be interacting with Arg209, Ala210, Lys323, Arg348, Lys351 Ser480, Val482 and Asn483 (Figure S10B), whereas in the case of the binding mode between subdomains IB, II and III, the GAG part of the molecule established interactions with Lys195, Lys199, Trp214, Arg218, Arg222, Arg257, Asn295, Ly436, Lys444 and Asp451 (Figure S10C). Notably, the principal component analysis of the MD trajectories indicated a concerted movement involving subdomains I and III, with subdomain III undergoing a hinge-like displacement towards subdomain I. Subdomain II remained comparatively stable, acting as a pivot point (Figure 6A). In particular, the calculated angle involving the three subdomains was around −4° (Figure 6B). This closure and opening of the subdomains I and III around _RE_GAG_2_ may imply a potential conformational selection mechanism, where _RE_GAG_2_ binding might be inducing or stabilizing distinct HSA conformations. The calculated free energies of binding further supported this hypothesis, as binding with the GAG moiety positioned along subdomains IB, II and IIIA in the closed conformation was more favorable (Table 2). In the case of _RE_GAG_1_, for the binding poses predicted by both GlycoTorch and Glide, the GAG moiety clustered at the same site, whereas the non-GAG moiety adopted distinct orientations, either at subdomain IIA at the FA7 site (predicted by GlycoTorch), or between subdomains IIA and IIIA (predicted by Glide) (Figure S5). The MD-refined structures showed interactions of the GAG moiety with residues Arg160, Lys181, Lys195, Lys432, Lys436, Lys439, His440, Lys444, and Tyr452, with the particularity that those predicted by Glide exhibited the biphenyl group as interacting preferentially with Lys195, Trp214, Arg218, Arg222, Val343, Val344, Pro447, Glu450, and Asp451 (Figure S10A). Furthermore, the calculated angles between HSA subdomains I, II and III throughout the MD trajectories in the presence of _RE_GAG_1_ also suggested a compact conformational state (Figure 6B). Overall, the observed recognition modes suggest that _RE_GAG_2_, in particular, may partially compete with warfarin at drug-binding site I, likely because of several residues shared between their interactions (*i.e.*, Phe211, Trp214 and Arg218).

#### 3.2.4. Subdomain III (FA5 Site)

Docking with GlycoTorch on the HSA structure PDB ID 1E7A predicted contacts of the biphenyl group of the _RE_GAG molecules with Phe502, Phe507, Leu532, His535 and Val576, while their respective GAG moieties were predicted to be solvent-exposed. In contrast, Glide did not predict any binding pose at the FA5 site (Figure S11). The obtained MD-based free energies of binding suggested particularly weak binding for _RE_GAG_1_ (ΔG_RE_GAG_1_ = −29.8 ± 8.4 kcal/mol; ΔG_RE_GAG_2_ = −39.6 ± 7.5 kcal/mol), and only little contacts between the GAG moieties and HSA residues Lys536, Lys538, Lys541 and Gln580 were observed. The predicted contacts of the biphenyl groups of both _RE_GAG molecules remained formed through the MD simulations (Figure S12).

#### 3.2.5. Cleft Between Subdomains I and III (FA9 Site)

**Figure 7:**
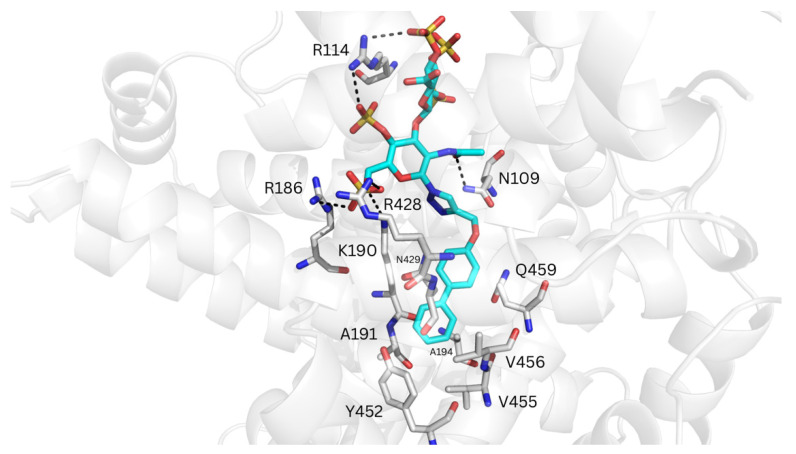
Representative MD-refined structure of _RE_GAG_2_ (in cyan sticks and colored by atom type) in complex with HSA (transparent gray cartoon) at the FA9 site. Relevant interacting HSA residues are shown in gray sticks and colored by atom type. H-bonds are depicted with dashed black lines. Figure generated with PyMOL v2.3.

Molecular docking of _RE_GAG_1_ and _RE_GAG_2_ with GlycoTorch and Glide at the FA9 site, using the HSA structure PDB ID 1E7A suggested a shared recognition site for the GAG moiety, with Arg114 and Arg186 acting as common interacting residues. However, for the biphenyl groups, two main recognition sites involving either Val456 at subdomain IIIA or around Phe507 and Phe509 at subdomain IIIB were predicted (Figure S11). The free energies of binding calculated from the representative structures generated by GlycoTorch indicated that _RE_GAG_2_ binds more favorably to the FA9 site of HSA than _RE_GAG_1_, with the biphenyl group oriented towards subdomain IIIA being energetically preferred with respect to its orientation towards subdomain IIIB (*i.e.*, ΔG_RE_GAG_1_ = −34.8 ± 11.6 kcal/mol, ΔG_RE_GAG_2_ = −44.1 ± 19.7 kcal/mol and ΔG_RE_GAG_1_ = −32.4 ± 4.1 kcal/mol, ΔG_RE_GAG_2_ = −40.1 ± 8.6 kcal/mol, respectively; Table 2). The GAG moiety in both _RE_GAG molecules formed H-bonds with Arg114, Arg186 and Lys190 at subdomain IB, Arg428 and Lys466 at subdomain IIIA, and Lys519 at subdomain IIIB. In the binding mode with the biphenyl group oriented towards subdomain IIIA, the GAG moieties also interacted with Asn109 and Arg145, while Lys190, Ala191, Ala194, Asn429 and Gln459 acted as anchor residues for the biphenyl groups. In the case of _RE_GAG_2_, the biphenyl group established additional van der Waals contacts with Val455 and Val456, as well as π-π interactions with Tyr452 (Figure 7, Figure S13). In the alternative recognition mode with the biphenyl groups oriented towards subdomain IIIB, the GAG moieties established additional electrostatic interactions with Arg117 and Lys524, whereas Phe509 served as the primary anchoring residue for the biphenyl groups. In _RE_GAG_1_, the biphenyl group also interacted with Phe507, Ala528 and Phe551, while in _RE_GAG_2_ it established additional van der Waals contacts with the side chain of Ile523 (Figure S14).

Finally, in order to evaluate the preferential recognition of _RE_GAG_1_ and _RE_GAG_2_ by HSA when the FA1–6 sites are occupied by fatty acids and warfarin is bound to the FA7 site, we performed additional molecular docking calculations using GlycoTorch and the HSA structure PDB ID 1H9Z, which contains all these mentioned ligands. The two _RE_GAG molecules were predicted to preferentially recognize the FA9 site. In addition, an alternative binding mode was predicted for both _RE_GAG molecules in which the GAG moiety was found interacting with the FA9 site and the biphenyl group oriented in the proximity of the heme site. Additionally, binding poses were also identified for _RE_GAG_2_ within the FA8 site (Figure S15). These findings are consistent with previous studies showing that drugs preferably recognize FA8 and FA9 sites when FA1-FA7 sites are occupied by long-chain fatty acids [61,62]. For instance, the monoterpene cantharidin was reported to bind to FA1 on ligand-free HSA, but to relocate to FA9 under FA-bound conditions [61].

### 3.3. Molecular Recognition of _RE_GAG by MSA

To facilitate the translation of preclinical studies in mice to human applications, a comprehensive comparison of ligand recognition by the human and mouse serum albumin proteins is essential. Consequently, a 3D model of MSA was built based on its homology to HSA by comparative modeling (see Methods Section 2.2, Figure S16) and assessed differences with the available MSA structure predicted by AlphaFold (AF-P02768-F1-v4) [35,36]. The N-terminal segment spanning Met1 to Ala26, which is missing from any available crystal structure, which exhibited very low prediction confidence (pLDDT < 50) in the AlphaFold model. Upon superimposition of the two 3D models (RMSD_Cα(27–608)_ = 2.0 Å), it was observed that the structural divergence between them primarily stems from subdomain I (Figure S16C). This reflects on the conformational plasticity of this subdomain and its variability in the different HSA templates available for the modeling (Figure 2B).

The MSA 3D model obtained by comparative modeling (see Methods) was used to predict interactions with _RE_GAG_1_ and _RE_GAG_2_. Interestingly, in contrast to the results obtained with HSA, in which the FA8 and FA9 sites were predicted as potential interacting sites, the _RE_GAG molecules were not predicted to bind at subdomain IA, the FA2, FA8 and the FA9 sites of MSA (Figure S17). The differences in shape and physicochemical properties between the FA8 and FA9 sites of MSA and HSA—particularly those involving residues Pro138, Leu214, Met222, Thr246, Leu464 and Arg456 in MSA (Arg114, Lys190, Leu198, Arg222, His440 and Gln459 in HSA)—may account for different ligand recognition profiles (Figure S18). In contrast to the results obtained for HSA, _RE_GAG_1_ and _RE_GAG_2_ might recognize drug-binding site II in MSA. These results are presented in detail in the following subsections:

#### 3.3.1. Subdomain IB (Drug Site III/Heme/FA1 Site)

MSA subdomain IB differs from that of HSA in the entrance to its cavity, particularly due to residues Pro138 and Phe139 in MSA, which correspond to Arg114 and Leu115 in HSA (Figure S16B). Nevertheless, the predicted binding poses for _RE_GAG_1_ and _RE_GAG_2_ using Glide and Glycotorch (Figure S17) broadly resembled those obtained for HSA in subdomain IB (Figure S2). For both _RE_GAG molecules, the GAG moiety formed H-bonds with Lys210 and Arg483. In addition, the biphenyl groups established π-π stacking interactions with Tyr162 and Tyr185. The calculated free energies of binding fell within a comparable range for both _RE_GAG molecules (Table 3). The MD-based interaction analysis further indicated extensive electrostatic interactions between the GAG moiety and residues Arg169, Lys210, Arg221 and, within subdomain III, Arg452, Arg456, Arg483, Lys490, and Lys543. In both _RE_GAG molecules, the biphenyl groups interacted with Tyr162 and Tyr185, and also established van der Waals contacts with the hydrophobic patch formed by Val166, Leu206 and Val209. In _RE_GAG_1_, the biphenyl group additionally interacted with Phe139 and Arg141, whereas in _RE_GAG_2_ π-π stacking interactions were observed with Tyr181 (Figure S19).

#### 3.3.2. Subdomain IIA (Drug-Binding Site I/Warfarin/FA7)

Subdomain IIA of MSA exhibits several physicochemical differences with respect to HSA. Notably, Arg222 and His242, which are key residues for warfarin binding in HSA, correspond to Thr246 and Asn266 in MSA. Similarly, Ile264 and Ala291, which were predicted to be crucial for _RE_GAG_2_ recognition by HSA, correspond to Met288 and Ser315 in MSA. Furthermore, Glu188 and Lys195 residues, situated at the entrance of the FA7 pocket in HSA, correspond to Lys212 and Arg219 in MSA. These alterations in residue composition are anticipated to alter the binding profile at this pocket. In this context, for instance, warfarin has been previously reported to bind weakly to MSA [63], in contrast to its strong binding to HSA [14,63].

Glide and Glycotorch predicted a single cluster for _RE_GAG_1_ within drug-binding site I (Figure S17). However, for _RE_GAG_2_, only GlycoTorch identified binding poses in this region. For both _RE_GAG, the GAG moieties were predicted to form H-bonds with Lys212, Arg219, Arg242, and Lys460, whereas the biphenyl groups were positioned within the hydrophobic region of the FA7 site. The analysis of the MD-refined structures indicated Lys460 as the strongest contributor to ligand binding (Figure S20). The biphenyl group of _RE_GAG_2_ was deeply disposed at the hydrophobic patch of the FA7 site, making van der Waals contacts with Leu243, Leu262, Leu284, Met288, Leu314 and Ser315. In _RE_GAG_1_, it was predicted to interact with Tyr174, Arg219, Gln220, Lys223, Cys269 and Arg281. Overall, the obtained results indicate that _RE_GAG_1_ exhibits more favorable free energy of binding than _RE_GAG_2_ at MSA drug-binding site I (Table 3).

#### 3.3.3. Subdomain IIB (FA6 Site)

The FA6 site is highly conserved between HSA and MSA. Glide and GlycoTorch predicted well-defined single clusters for _RE_GAG_1_ within this site, whereas only two disperse binding poses were observed for _RE_GAG_2_ (Figure S17). The biphenyl moiety of _RE_GAG_1_ was predicted to interact with Arg233. Furthermore, the sulfate groups of the GAG moiety were predicted by Glide to establish H-bonds with Lys236 and Lys257 (Figure S17B). Overall, the predicted binding poses closely resembled those obtained with Glide for HSA (Figure S5B). Thus, no further MD refinement was pursued.

#### 3.3.4. Subdomain IIIA (Drug-Binding Site II)

**Figure 8:**
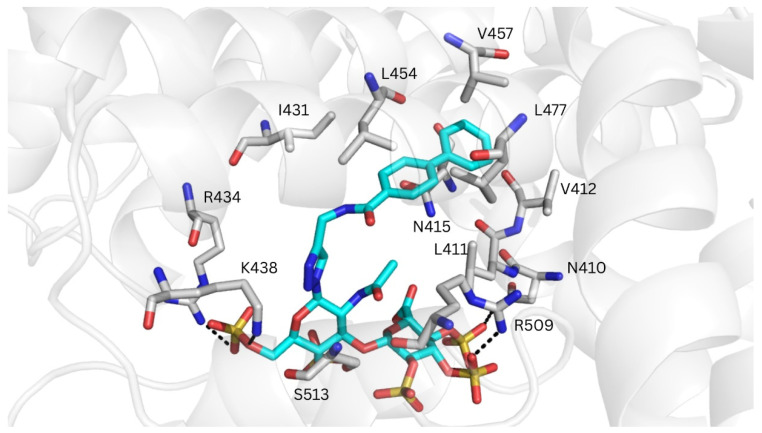
Representative MD-refined structure of _RE_GAG_1_ (in cyan sticks and colored by atom type) in complex with MSA (transparent gray cartoon) at drug-binding site II. Relevant interacting MSA residues are shown in gray sticks and colored by atom type. H-bonds are depicted with dashed black lines. Figure generated with PyMOL v2.3.

Only GlycoTorch predicted binding poses for _RE_GAG_1_ at drug-binding site II, with the biphenyl group positioned within this region and the GAG moiety forming H-bonds with Lys438 (Figure S17A). This markedly differs from the results obtained for HSA, with no _RE_GAG binding poses identified at this site. A plausible explanation for the distinct binding profiles observed in MSA may arise from differences at the entrance of the binding cavity, where the GAG moieties were located. In particular, Thr414 and Thr516 in MSA—corresponding to Gln390 and Glu492 in HSA—appear to make drug-binding site II more accessible. In addition, the size of the binding pocket in MSA seems wider than in HSA (Figure S18B), a feature that may be determinant for accommodating molecules of the size of the investigated _RE_GAG molecules. The MD-refined structures showed extensive electrostatic interactions between the GAG moiety and Arg372, Lys375, Arg434, Lys438, and Arg509, and H-bonding with Asn410 and Ser513. In addition, the biphenyl groups made van der Waals contacts with Leu411, Val412, Asn415, Ile431, Leu454, Val457 and Leu477 (Figure 8). Interestingly, this recognition mode resembles those previously reported for commonly administered drugs that target site II of HSA. For instance, propofol is known to make hydrophobic interactions with Leu387, Ile388, Asn391, Leu407, and Val433, and a hydrogen bond with the main chain of Leu430 (corresponding to Leu411, Ile412, Asn415, Leu431, Val457, and Leu454 in MSA) [64]. In contrast, diazepam, diflunisal and ibuprofen exhibit a slightly different binding mode at drug-binding site II, primarily interacting with residues Leu387, Asn391, and Leu453 in HSA [13] (corresponding to Leu411, Asn415 and Leu477 in MSA).

#### 3.3.5. Subdomain IIIB (FA5 Site)

The core residues at the binding pocket of subdomain IIIB are highly conserved between HSA and MSA. Interestingly, a single significant difference is Lys402 in HSA, which corresponds to Gly426 in MSA (Figure S16B). This residue variation might be relevant for differences in the recognition of the GAG moieties. Only GlycoTorch predicted one cluster for each _RE_GAG_1_ molecule at the back side of subdomain IIIB in MSA (Figure S17), with their biphenyl group overlapping with the FA5 site. In particular, the biphenyl groups were predicted establishing π-π interactions with Phe575, and the GAG moieties forming H-bonds with Lys549 (Figure S17A).

The MD-refined structures indicated that Lys421 (subdomain IIIA), Lys545, Lys549 and Lys569 constitute the anchor recognition residues for the GAG moieties. Surrounding this positively charged patch, the biphenyl groups established van der Waals interactions with Phe531, Ala552, Leu553, Leu556, Met572 and Phe575, which form a deep hydrophobic cleft (Figure S21). In _RE_GAG_2_, the biphenyl group showed additional interactions with Val571 and Leu599.

## 4. Discussion

The biological activity of a ligand depends not only on its interactions with its target molecule but also on its binding to plasma proteins. Investigating ligand binding to proteins such as human serum albumin provides valuable insights into their pharmacokinetics and potential pharmacological effects [21]. Furthermore, assessing ligand competition for drug binding sites or co-binding is essential for understanding possible interactions in vivo and their therapeutic effect [65,66,67,68].

HSA exhibits a large ligand-binding capacity. Among the nine binding sites for drugs and other ligands so-far described in HSA, FA1 (drug site III or heme-binding site), FA3-FA4 (drug site II) and FA7 (drug site I) are generally considered the most relevant for drug binding [11]. In this work, the two investigated _RE_GAG molecules were predicted to preferentially recognize HSA drug-binding sites III and I, and, in the particular case of _RE_GAG_2_, also sites FA8 and FA9. Notably, no interactions were observed for any of the _RE_GAG molecules at drug-binding site II.

Drug-binding site III in HSA is characterized by a hydrophobic D-shaped cavity formed by residues Tyr138 and Tyr161, which serves as the recognition site for heme. For instance, the monoterpene cantharidin was reported to bind in this cavity on ligand-free HSA through hydrophobic interactions with the aromatic rings of Tyr138 and Tyr161, and it was suggested to inhibit competitively heme-Fe(III) association [61]. Our docking studies predicted comparable binding affinities for the _RE_GAG molecules and cantharidin, suggesting that the _RE_GAG molecules might compete with other drugs for this site under HSA ligand-free conditions. In addition, the free energies of binding obtained along the MD trajectories suggest strong binding of the _RE_GAG molecules to the HSA heme site, especially when compared to the values reported for cantharidin at the FA1 site [61]. The _RE_GAG molecules not only recognized the two key Tyr residues for heme binding, but also relevant basic residues disposed between subdomain IB and IIIA. Furthermore, we observed an opening movement between the helices containing Tyr138 and Tyr161 during _RE_GAG recognition, a phenomenon previously associated with allosteric transitions in the B-to-N state switch of HSA [69]. It could be hypothesized that such conformational change corresponds to a general feature of HSA’s dynamic response, not limited to pH or fatty acid binding.

Interactive association of drugs binding to HSA typically occurs at drug-binding site I, which has a large hydrophobic cavity capable of accommodating multiple drugs simultaneously. At this site, HSA generally shows a preference for binding anionic over cationic drugs [70,71]. For instance, the anticoagulant warfarin, the antirheumatic drugs azapropazone and indomethacin, the antibiotic sulfisoxazole, the contrast agent iodipamide and the diuretic furosemide have been reported to bind drug-binding site I on HSA. Studies from Yang et al. [72] and Peng et al. [73] also reported strong binding of hydroxylated polybrominated diphenyl ethers (*K*_a_ values between 0.5–1.8 × 10^7^ M^−1^) and perfluorononanonic acid (*K*_a_ = 7.8 × 10^6^ M^−1^) to the subdomain IIA. Interestingly, their calculated free energies of binding were less favorable than those obtained for the _RE_GAG molecules, which may suggest strong binding of _RE_GAG to the FA7 site. Furthermore, co-binding of fatty acids and drugs has also been documented [13,67,74], although it has been reported that fatty acids weakly bind to subdomain IIA and can be replaced by drugs [75,76]. For instance, molecules like AZT (3′-Azido-3′-deoxythymidine) are known to coexist with fatty acids in the IIA subdomain. The presence of a fatty acid in the FA7 site can lead to AZT binding to a new subsite within this subdomain, where it forms a hydrogen bond with the fatty acid itself. This new subsite is besieged by hydrophilic and polar amino acids, including Glu153, Ser192, Lys195, and Gln196 from subdomain IB and Lys199, His242, Arg257, and Glu292 from site I [77]. Both investigated _RE_GAG molecules were predicted to interact with several of those residues (*i.e.*, Gln196, Lys199, His242 and Arg257). In general, displacement interactions become clinically relevant only for drugs that are highly bound to HSA (>90%, [78]), such as warfarin, phenytoin, or diazepam. In contrast, drugs with plasma protein binding of less than 85% do not raise concerns for adverse drug-drug interactions [79]. Although the predicted free energies of binding for the _RE_GAG are not directly comparable to experimental binding affinities, they might be considered within the affinity range reported for typical therapeutic ligands of HSA [14,21,72,80,81]. Based on our observations, the possibility of _RE_GAG_1_ and _RE_GAG_2_ interacting with or displacing certain drugs in drug-binding site I should not be excluded. Therefore, the co-administration of _RE_GAG_1_ and _RE_GAG_2_ with other drugs should be further investigated experimentally in future studies and, if necessary, carefully considered to mitigate contraindications [65,66,67,68].

The FA8 site and, to a lesser extent, FA9 were also identified in our studies as potential binding sites, particularly for _RE_GAG_2_. Interestingly, FA8 and FA9 have been reported as additional ligand binding pockets becoming accessible when long-chain fatty acids such as myristic acid occupy the FA1-FA7 sites [11,15,16]. For instance, earlier docking studies revealed ligand recognition at the FA8 and FA9 sites in the presence of endogenous molecules such as heme-Fe(III) and long-chain FA [61,62]. Furthermore, FA9 has been previously identified as the binding site for thyroxine, and has been referred to as thyroxine binding site Tr5 [15]. Both FA8 and FA9 sites are formed by fatty acid-induced conformational changes in the crevice between IA-IB-IIA subdomains on one side and IIB-IIIA-IIIB subdomains on the other side [16,74,82]. The docking results obtained with _RE_GAG_1_ and _RE_GAG_2_ on the structure of HSA with bound fatty acids and warfarin (PDB ID 1H9Z) suggested that the _RE_GAG molecules might be preferentially associate to the FA8 and FA9 sites in the presence of such competing ligands. Given that HSA primarily acts as a carrier of fatty acids and heme in blood [11], the extent to which the investigated _RE_GAG molecules can bind to FA1, FA5, FA6, or drug-binding site I may be competitively or allosterically influenced by these endogenous ligands. This could potentially lead to an increased unbound fraction of those ligands in plasma. This possibility underscores the importance of carefully revising the optimal dosing of _RE_GAG_1_ and _RE_GAG_2_. Likewise, physiological conditions affecting HSA plasma levels (e.g., analbuminemia, dehydration) may also impact the pharmacokinetics and pharmacodynamics of the investigated _RE_GAG molecules [83].

Regarding the FA5/6 and FA8/9 sites, _RE_GAG_2_ generally displayed more favorable binding free energies than _RE_GAG_1_ when being docked to the ligand-free HSA. These results can be attributable to the different chemical composition of their respective linker group joining the non-GAG and GAG moieties, affecting the conformational adaptability of the molecule. In particular, the ether linker in _RE_GAG_2_ is inherently more flexible than the rotationally restricted amide linker in _RE_GAG_1_. Given that the FA6 and FA8/9 sites are more solvent-accessible than other fatty acid sites and involve several flexible loop regions, the conformational adaptability of _RE_GAG_2_ might facilitate its ability to adopt conformations that fit more snugly into various albumin binding pockets.

With respect to the assessment of binding of _RE_GAG_1_ and _RE_GAG_2_ to murine serum albumin, our results suggested a similar recognition to the predicted for the human protein, particularly concerning the occupation of the most conserved sites among the homologs (*i.e.*, FA1, FA6, and FA7). Nevertheless, subtle sequence differences between HSA and MSA resulted in different predicted accessibility to certain binding sites. For instance, _RE_GAG_1_ was found to bind in drug-binding site II in MSA but not in HSA, which could be due to the fact that this site differs in the size of the entrance of the binding cavity in both proteins.

When comparing _RE_GAG_1_ and _RE_GAG_2_ binding to HSA and MSA, additional recognition differences were observed. In particular, binding of _RE_GAG_1_ was predicted to be slightly more favorable to the FA7 site in HSA compared to _RE_GAG_2_, whereas a greater preference for recognition of _RE_GAG_1_ at this site was notable in MSA. These observations might be attributed to alterations in the shape of the binding pocket, arising from variations between the two proteins in the physicochemical properties of residues within the FA7 site and its entrance.

While the present work provides valuable insights based on in silico predictions, the results obtained should be interpreted with caution. Future experimental work will be essential to confirm our predictive models and to further substantiate the conclusions drawn from them.

## 5. Conclusions

Overall, this work represents a comprehensive study investigating, at the atomic level, the serum albumin binding properties of two novel bone-regenerative lead molecules currently undergoing preclinical testing. Our investigations delve into the dynamic mechanisms of molecular recognition suggesting that these molecules could strongly bind to serum albumin and induce conformational changes. Furthermore, they could potentially co-exist or even compete for known recognition sites of endogenous ligands or other drugs. Our mechanistic studies also include a comparative analysis between the serum albumin human and murine proteins, which is of particular relevance for translating the knowledge from preclinical studies in mice to future human applications. Therefore, the results obtained here provide valuable insights for advancing these novel _RE_GAG molecules to the next stage of pharmacological profiling, which is essential for their potential clinical application and for establishing a rational basis for future molecular design aimed at improving pharmacokinetic properties.

## Figures and Tables

**Table 1:** Predicted plasma protein binding (given in %) for _RE_GAG_1_ and _RE_GAG_2_.

Molecule	ADMET-AI	ADMETlab	Deep-PK	AdmetSAR
_RE_GAG_1_	67.69	61.10	34.24	77.80
_RE_GAG_2_	70.11	83.75	83.89	76.06

**Table 2:** Free energies of binding of _RE_GAG molecules to HSA. Energies were obtained with MM-GBSA from three independent 100 ns MD simulations.

HSA Ligand Binding Sites	ΔG_RE_GAG_1_ (kcal/mol) ^a^	ΔG_RE_GAG_2_ (kcal/mol) ^a^
Subdomain IB (FA1 site)	−52.8 ± 1.6	−56.6 ± 4.6
Subdomain II (FA7 site)	−57.9 ± 10.2 ^b^	−49.1 ± 9.8 ^c^
Subdomain II (FA6 site)	−23.2 ± 1.9	−38.4 ± 8.9
Subdomain II (FA8 site)	−33.4 ± 3.9	−53.1 ± 4.5 ^d^
Subdomain III (FA5 site)	−29.8 ± 8.4	−39.6 ± 7.5
Cleft between subdomains I and III (FA9 site)	−34.8 ± 11.6 ^e, f^	−44.1 ± 19.7 ^e, g^

^a^ Values represent the mean ± SD from three independent 100 ns MD simulations. ^b^ Value obtained for the most favorable MD-refined complexes from GlycoTorch predictions and the structure of HSA from PDB ID 1H9Z. For the less favorable MD-refined complexes was ΔG = −39.3 ± 7.3. The value obtained when using PDB ID 1N5U was ΔG = −47.6 ± 1.2. ^c^ Value obtained for MD-refined complexes from GlycoTorch and HSA from PDB ID 1H9Z. The value obtained when using PDB ID 1N5U was ΔG = −47.5 ± 14.2. ^d^ Value obtained for MD-refined complexes from Glide with the GAG moiety oriented between subdomains IB, II and III. The value obtained for MD-refined complexes from GlycoTorch with the GAG moiety at the HSA surface was ΔG = −35.4 ± 8.2. ^e^ _RE_GAG binding modes with the biphenyl groups oriented towards subdomain IIIA. ^f^ The value for the alternative binding mode with the biphenyl group oriented towards subdomain IIIB was ΔG = −32.4 ± 4.1. ^g^ The value obtained for the alternative binding mode with the biphenyl group oriented towards the subdomain IIIB was ΔG = −40.1 ± 8.6.

**Table 3:** Free energies of binding of _RE_GAG molecules to MSA. Energies were obtained with MM-GBSA from three independent 100 ns MD simulations.

MSA Ligand Binding Sites	ΔG _RE_GAG_1_ (kcal/mol) ^a^	ΔG _RE_GAG_2_ (kcal/mol) ^a^
Subdomain IB (FA1 site)	−41.5 ± 12.6	−44.3 ± 11.1
Subdomain II (FA7 site)	−31.1 ± 3.0	−19.6 ± 10.2
Subdomain II (FA3/4 site)	−24.3 ± 7.1	---
Subdomain III (FA5)	−28.1 ± 4.0	−36.1 ± 4.5

^a^ Values represent the mean ± SD from three independent 100 ns MD simulations.

## Data Availability

The original contributions presented in this study are included in the article/Supplementary Material. Further inquiries can be directed to the corresponding authors.

## Supplementary Materials

The following supporting information can be downloaded at: https://www.mdpi.com/article/10.3390/pharmaceutics17111445/s1, Table S1: Structural comparison of HSA crystallographic structures: RMSD analysis; Figure S1: Docking results obtained with Glide for HSA-binding ligands in comparison to experimentally determined HSA-ligand complex structures; Figure S2: Molecular recognition of _RE_GAG molecules by HSA. Docking results of _RE_GAG_1_ (cyan sticks) and _RE_GAG_2_ (azure sticks) within two distinct binding sites on HSA (PDB ID 1N5U, grey cartoon) using (A) GlycoTorch and (B) Glide; Figure S3: RMSD for _RE_GAG molecules interaction with HSA from PDB ID 1N5U at the FA1 site. Values are relative to the first frame of the MD simulation and without structural fitting; Figure S4: MD-analysis of angles involving subdomains IA, IIB and IIIA of HSA when complexed with _RE_GAG molecules at the FA1 site. Violin representation of calculated angles from 100 ns MD simulations defined by the C_α_ carbon of residues P110, A364 and S427 of HSA from PDB ID 1N5U; Figure S5: Molecular recognition of _RE_GAG molecules by HSA. Docking results of _RE_GAG_1_ (cyan sticks) and _RE_GAG_2_ (azure sticks) with HSA (PDB ID 1H9Z, grey cartoon) using (A) GlycoTorch and (B) Glide; Figure S6: Molecular recognition of _RE_GAG molecules by HSA. Docking results of _RE_GAG_1_ (cyan sticks) and _RE_GAG_2_ (azure sticks) with HSA (PDB ID 1H9Z, grey cartoon) using AutoDock3; Figure S7: Pairwise binding free energy contributions calculated with MM-GBSA from three independent 100 ns MD simulations of HSA (from PDB ID 1H9Z) in complex with (A)-(B) _RE_GAG_1_ and (C) _RE_GAG_2_ bound to the warfarin (FA7) site; Figure S8: Pairwise binding free energy contributions calculated with MM-GBSA from three independent 100 ns MD simulations of HSA (from PDB ID 1N5U) in complex with (A) _RE_GAG_1_ and (B) _RE_GAG_2_ bound to the warfarin (FA7) site; Figure S9: Pairwise binding free energy contributions calculated with MM-GBSA from three independent 100 ns MD simulations of HSA (from PDB ID 1H9Z) in complex with (A) _RE_GAG_1_ and (B) _RE_GAG_2_ bound to the FA6 site; Figure S10: Pairwise binding free energy contributions calculated with MM-GBSA from three independent 100 ns MD simulations of HSA (from PDB ID 1H9Z) in complex with (A) _RE_GAG_1_ and (B)-(C) _RE_GAG_2_ bound to the FA8 site; Figure S11: Molecular recognition of _RE_GAG molecules by HSA. Docking results of _RE_GAG1 (cyan sticks) and _RE_GAG_2_ (azure sticks) with HSA (PDB ID 1E7A, grey cartoon, back side view) using (A) GlycoTorch and (B) Glide; Figure S12: Pairwise binding free energy contributions calculated with MM-GBSA from three independent 100 ns MD simulations of HSA (from PDB ID 1E7A) in complex with (A) _RE_GAG_1_ and (B) _RE_GAG_2_ bound to the FA5 site; Figure S13: Pairwise binding free energy contributions calculated with MM-GBSA from three independent 100 ns MD simulations of HSA (from PDB ID 1E7A) in complex with (A) _RE_GAG_1_ and (B) _RE_GAG_2_ bound to the FA9 site. The results shown correspond to complexes in which the biphenyl groups of _RE_GAG molecules are oriented towards the subdomain IIIA; Figure S14: Pairwise binding free energy contributions calculated with MM-GBSA from three independent 100 ns MD simulations of HSA (from PDB ID 1E7A) in complex with (A) _RE_GAG_1_ and (B) _RE_GAG_2_ bound to the FA9 site. The results shown correspond to complexes in which the biphenyl groups of _RE_GAG molecules are oriented towards the subdomain IIIB; Figure S15: Molecular recognition of _RE_GAG molecules by HSA. Docking results of _RE_GAG_1_ (cyan sticks) and _RE_GAG_2_ (azure sticks) in warfarin- (red-brown sticks and transparent surface) and FA-bound (purple sticks and transparent surface) HSA (PDB ID 1H9Z, grey cartoon) using GlycoTorch; Figure S16: Molecular modeling of MSA; Figure S17: Molecular recognition of _RE_GAG molecules by MSA. Docking results of _RE_GAG_1_ (cyan sticks) and _RE_GAG_2_ (azure sticks) with the MSA model using (A) GlycoTorch and (B) Glide; Figure S18: Comparative of HSA (left, in grey) and MSA (right, in magenta) recognition sites (shown as molecular surfaces). Non-conserved residues between the human and murine proteins are labeled; Figure S19: Pairwise binding free energy contributions calculated with MM-GBSA from three independent 100 ns MD simulations of MSA in complex with (A) _RE_GAG_1_ and (B) _RE_GAG_2_ bound to the FA1 site; Figure S20: Pairwise binding free energy contributions calculated with MM-GBSA from three independent 100 ns MD simulations of MSA in complex with (A) _RE_GAG_1_ and (B) _RE_GAG_2_ bound to the FA7 site; Figure S21: Pairwise binding free energy contributions calculated with MM-GBSA from three independent 100 ns MD simulations of MSA in complex with (A) _RE_GAG_1_ and (B) _RE_GAG_2_ bound to the FA5 site.

## Abbreviations

ADMET	Absorption, Distribution, Metabolism, Excretion, and Toxicity
FA	Fatty Acid
HSA	Human Serum Albumin
MD	Molecular Dynamics
MSA	Murine Serum Albumin
