# Supplementary material for: In Silico Analysis of Serum Albumin Binding by Bone-Regenerative Hyaluronan-Based Molecules
Source: Pharmaceutics. 2025 Nov 8;17(11):1445. doi: 10.3390/pharmaceutics17111445 (PMC12655611; doi:10.3390/pharmaceutics17111445)
Supplement: Supplementary file 1 [file pharmaceutics-17-01445-s001.zip › pharmaceutics-3906405-supplementary.pdf]

pharmaceutics

MDPI

List of contents

Table S1 ....S3
Figure S1 ....S4
Figure S2 ....S5
Figure S3 ....S6
Figure S4 ....S6
Figure S5 ....S7
Figure S6 ....S8
Figure S7 ....S9
Figure S8 ....S10
Figure S9 ....S11
Figure S10 ....S12
Figure S11 ....S13
Figure S12 ....S14
Figure S13 ....S15
Figure S14 ....S16
Figure S15 ....S17
Figure S16 ....S18
Figure S17 ....S19
Figure S18 ....S20
Figure S19 ....S21
Figure S20 ....S22
Figure S21 ....S23

pharmaceutics

Table S1. Structural comparison of HSA crystallographic structures: RMSD analysis.

HSA PDB IDs	Global RMSD$_{C\alpha}$	Local RMSD$_{C\alpha}$		
		Subdomain I	Subdomain II	Subdomain III
1H9Z - 1N5U	0.6	0.4	0.4	0.4
1E7A - 1H9Z	2.9	1.1	0.4	1.5
1E7A - 1N5U	3.0	1.2	0.6	1.5

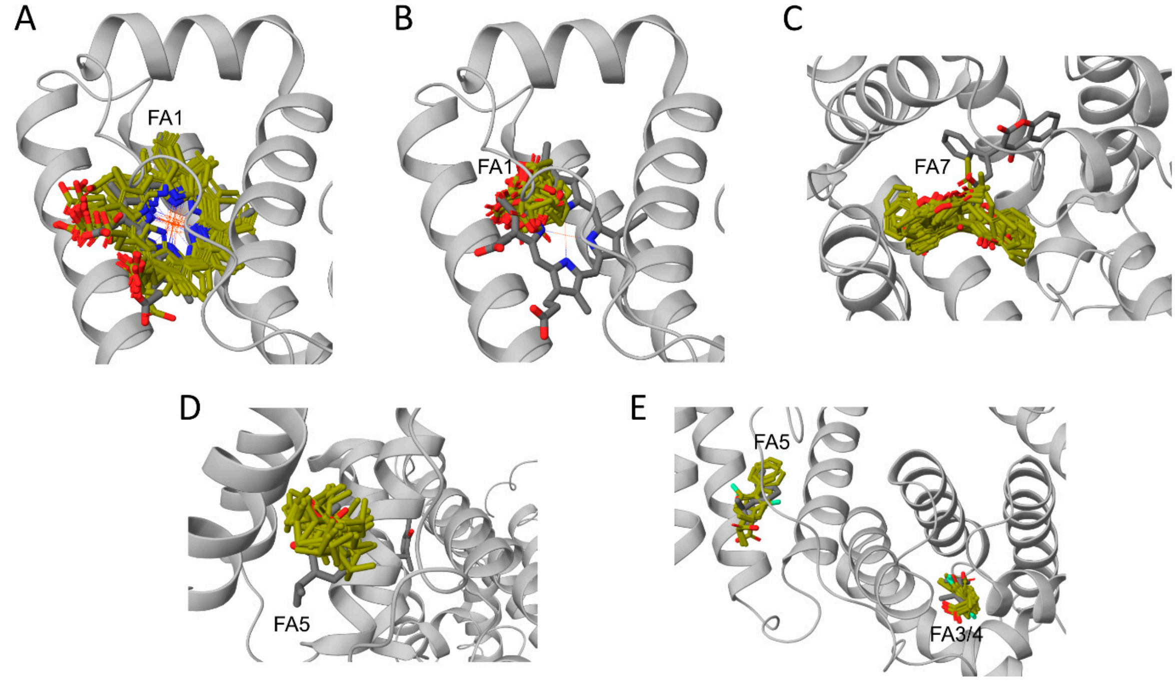

Figure S1. Docking results obtained with Glide for HSA-binding ligands in comparison to experimentally determined HSA-ligand complex structures. HSA is represented by a grey cartoon. Ligand binding poses obtained by docking are shown in green sticks, and the reference crystallographic ligand structures are shown in dark grey sticks. (**A**) Heme-Fe(III) (PDB ID 1N5U), (**B**) cantharidin (PDB ID 1N5U), (**C**) warfarin (PDB ID 1H9Z), (**D**) propofol (PDB ID 1E7A) and (**E**) flurbiprofen (PDB ID 1E7A). Figure generated with Maestro v14.3.

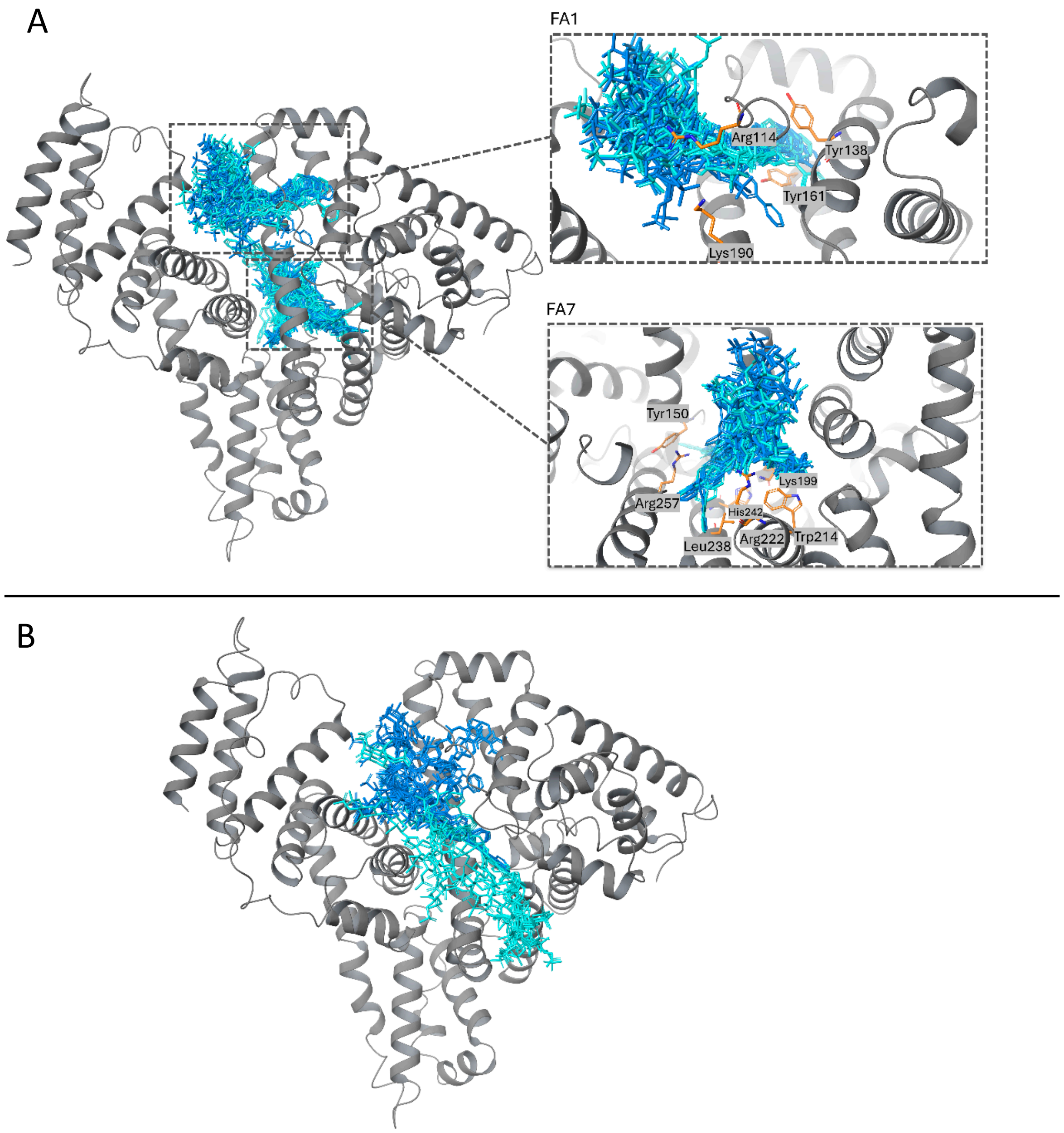

Figure S2. Molecular recognition of reGAG molecules by HSA. Docking results of reGAG$_1$ (cyan sticks) and reGAG$_2$ (azure sticks) within two distinct binding sites on HSA (PDB ID 1N5U, grey cartoon) using (**A**) GlycoTorch and (**B**) Glide. In (**A**), zoom in of the FA1 (heme-Fe(III)-binding) and FA7 (drug-binding site I) sites. Relevant recognition residues are displayed in orange sticks and labelled. Figure generated with Maestro v14.3.

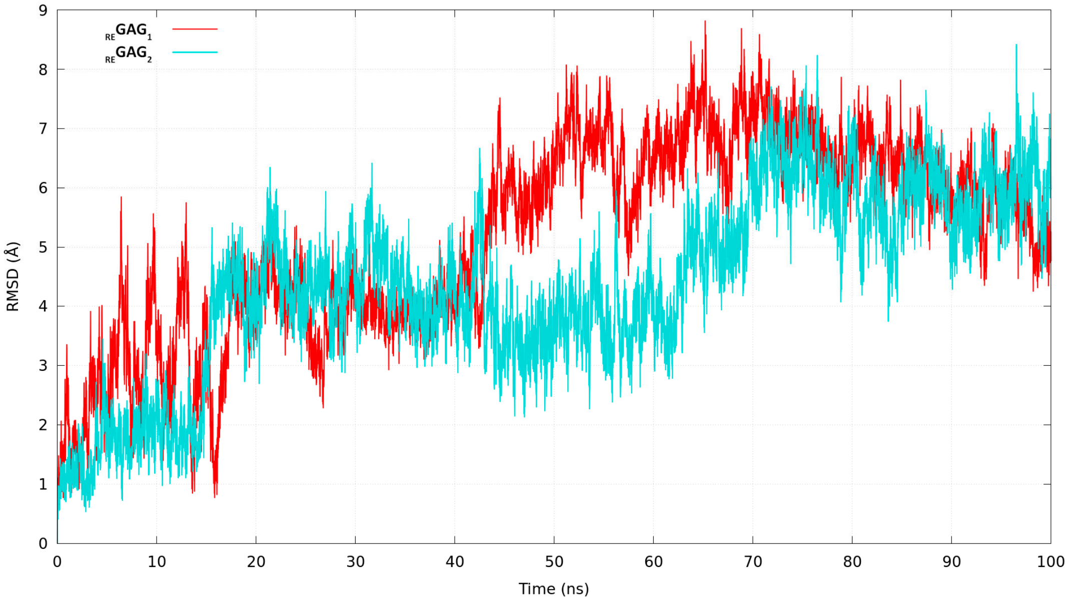

Figure S3. RMSD for the interaction of the REGAG molecules with HSA (PDB ID 1N5U) at the FA1 site. Values are relative to the first frame of the MD simulation and without structural fitting of the REGAG molecule.

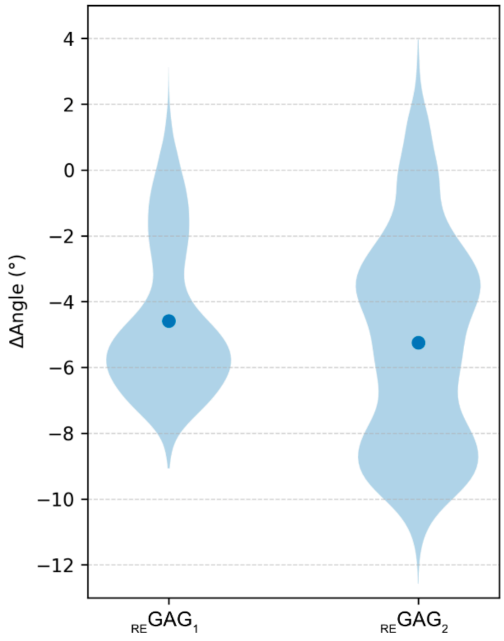

Figure S4. MD-analysis of angles involving subdomains IA, IIB and IIIA of HSA when complexed with the REGAG molecules at the FA1 site. Violin representation of calculated angles from 100 ns MD simulations defined by the Cα carbon of residues P110, A364 and S427 of HSA (PDB ID 1N5U). ΔAngle values

correspond to the difference between the calculated angles over the MD trajectories and the angle from the initial structure. The mean is highlighted by a blue dot.

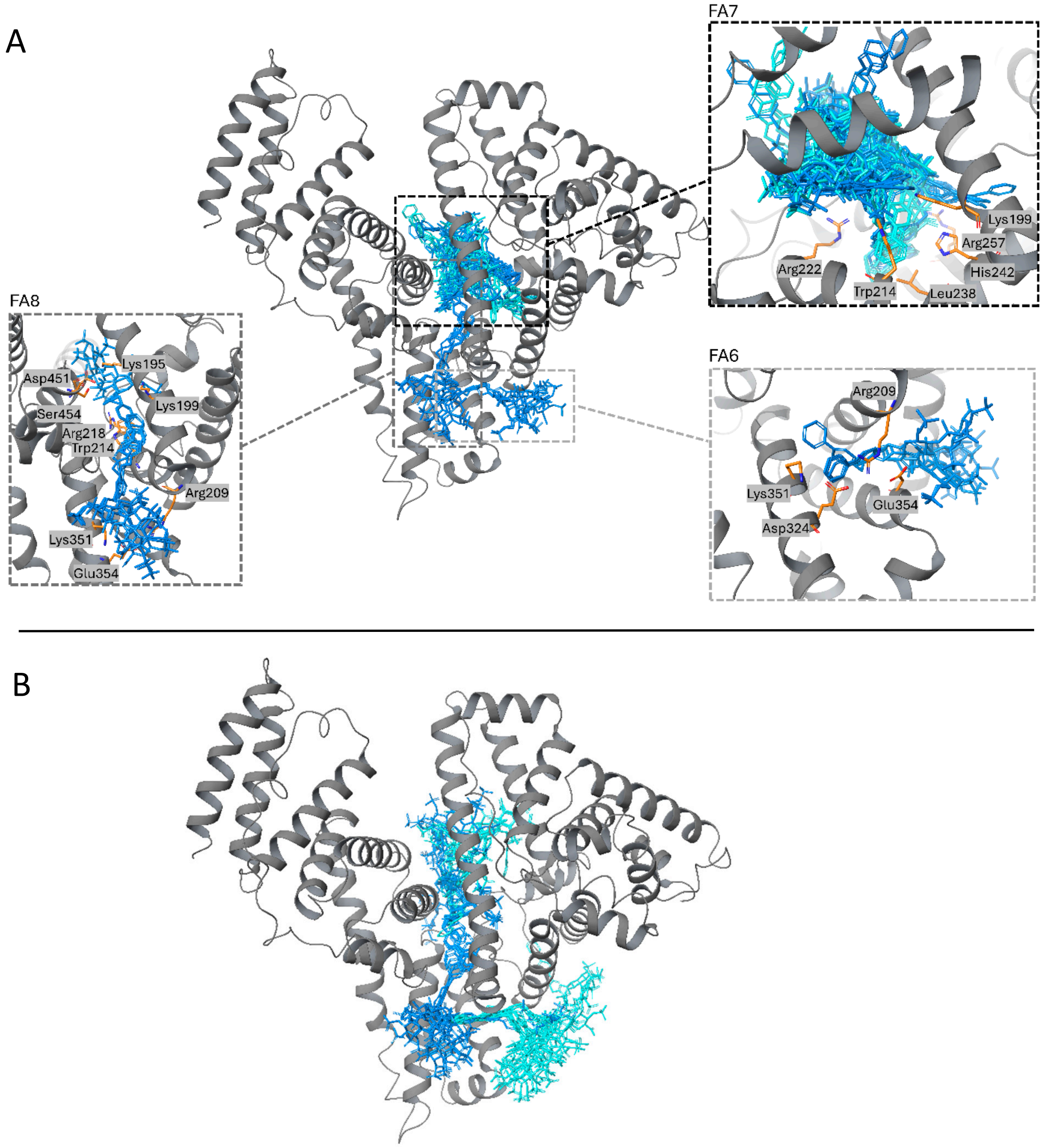

Figure S5. Molecular recognition of REGAG molecules by HSA. Docking results of REGAG1 (cyan sticks) and REGAG2 (azure sticks) with HSA (PDB ID 1H9Z, grey cartoon) using (**A**) GlycoTorch and (**B**) Glide. In (A) zoom in of binding poses of the REGAG molecules within three distinct binding sites on HSA: FA7 (drug-

binding site I), FA6 and FA8. Relevant recognition residues are displayed in orange sticks and labelled. Figure generated with Maestro v14.3.

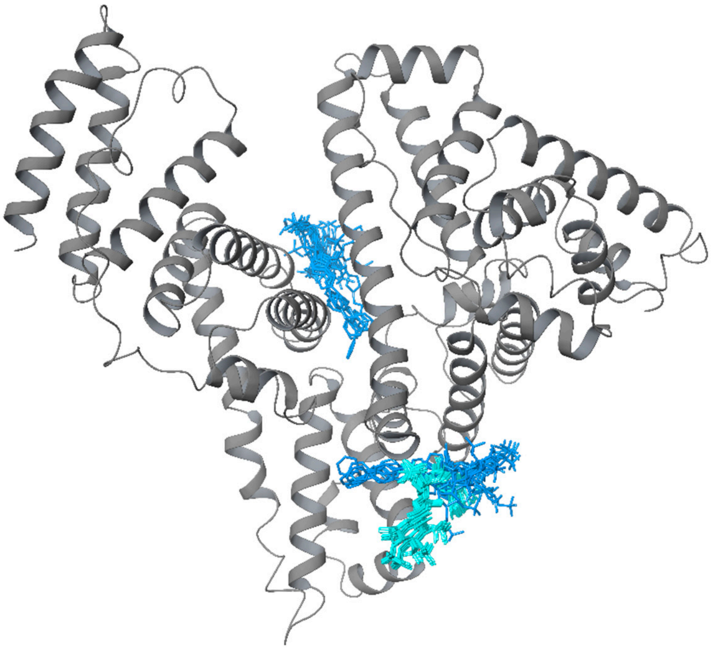

Figure S6. Molecular recognition of REGAG molecules by HSA. Docking results of REGAG1 (cyan sticks) and REGAG2 (azure sticks) with HSA (PDB ID 1H9Z, grey cartoon) using AutoDock3. Figure generated with Maestro v14.3.

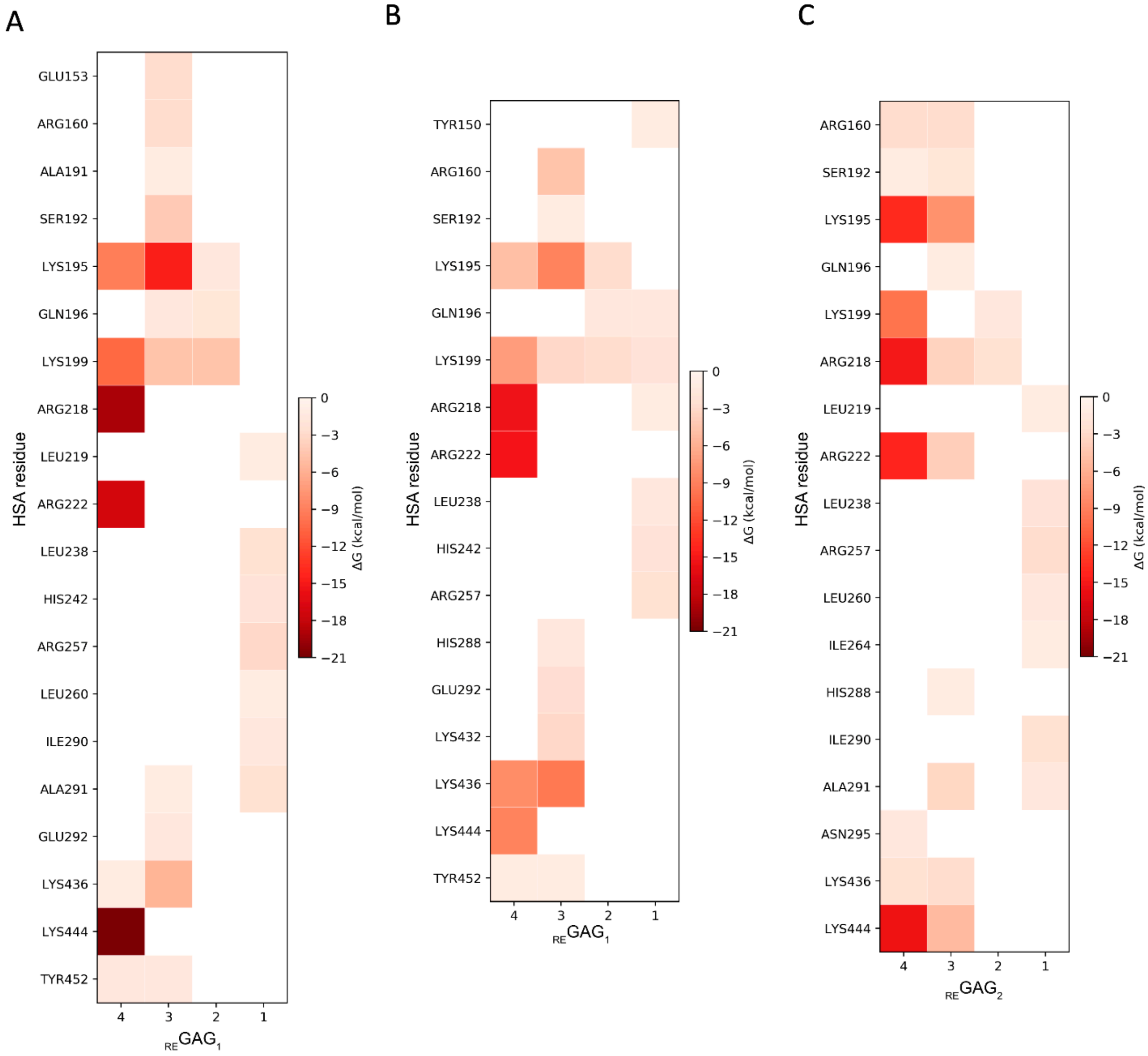

Figure S7. Pairwise binding free energy contributions calculated with MM-GBSA from three independent 100 ns MD simulations of HSA (PDB ID 1H9Z) in complex with (**A**)-(**B**) REGAG1 and (**C**) REGAG2 bound to the warfarin (FA7) site. The interacting energies of the most favorable (**A**) and the less favorable (**B**) binding mode of REGAG1 by HSA are shown. Mean values are indicated by the gradient-colored side bar. The different fragments of each REGAG molecule are represented in the x axis by the numbers: (1) biphenyl group, (**2**) linker, (3) fully sulfated N-acetylglucosamine (GlcNAc), and (4) fully sulfated glucuronic acid (GlcA).

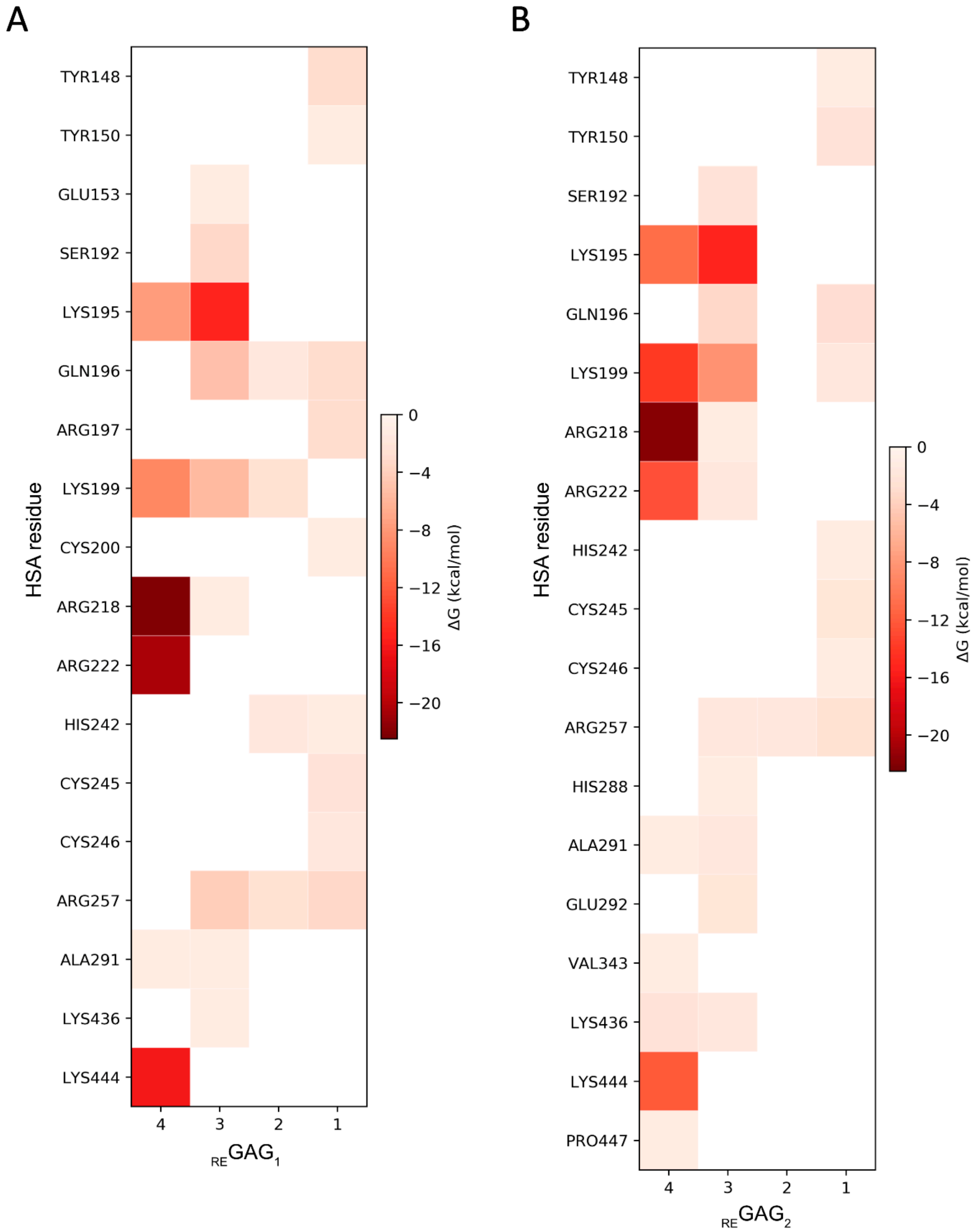

Figure S8. Pairwise binding free energy contributions calculated with MM-GBSA from three independent 100 ns MD simulations of HSA (PDB ID 1N5U) in complex with (**A**) REGAG1 and (**B**) REGAG2 bound to the warfarin site (FA7). Mean values are indicated by the gradient-colored side bar. The different fragments of each REGAG molecule are represented in the *x* axis by the numbers: (1) biphenyl group, (2) linker, (3) fully sulfated N-acetylglucosamine (GlcNAc), and (4) fully sulfated glucuronic acid (GlcA).

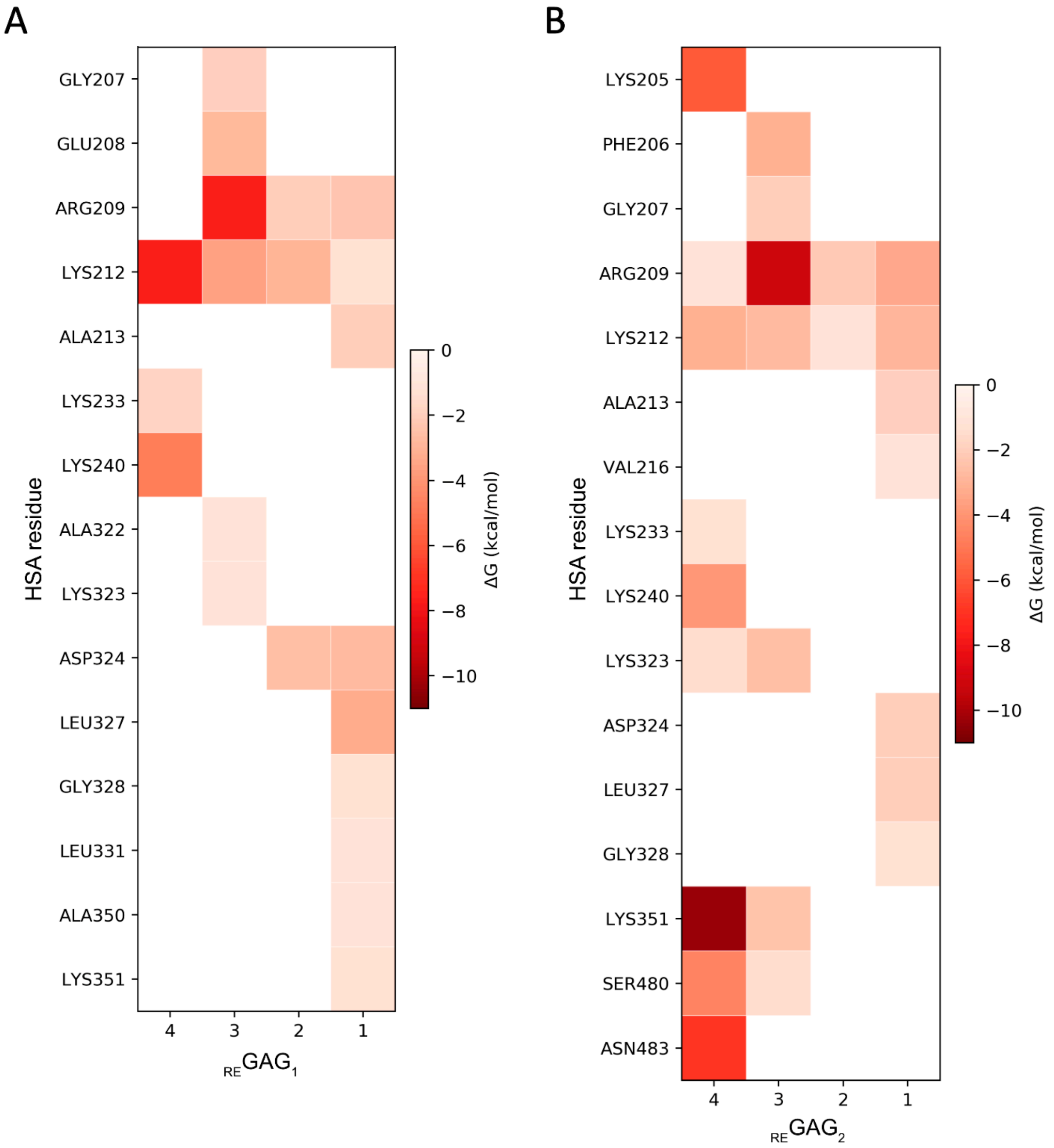

Figure S9. Pairwise binding free energy contributions calculated with MM-GBSA from three independent 100 ns MD simulations of HSA (PDB ID 1H9Z) in complex with (**A**) $_{RE}GAG_1$ and (**B**) $_{RE}GAG_2$ bound to the FA6 site. Mean values are indicated by the gradient-colored side bar. The different fragments of each $_{RE}$GAG molecule are represented in the x axis by the numbers: (1) biphenyl group, (2) linker, (3) fully sulfated N-acetylglucosamine (GlcNAc), and (4) fully sulfated glucuronic acid (GlcA).

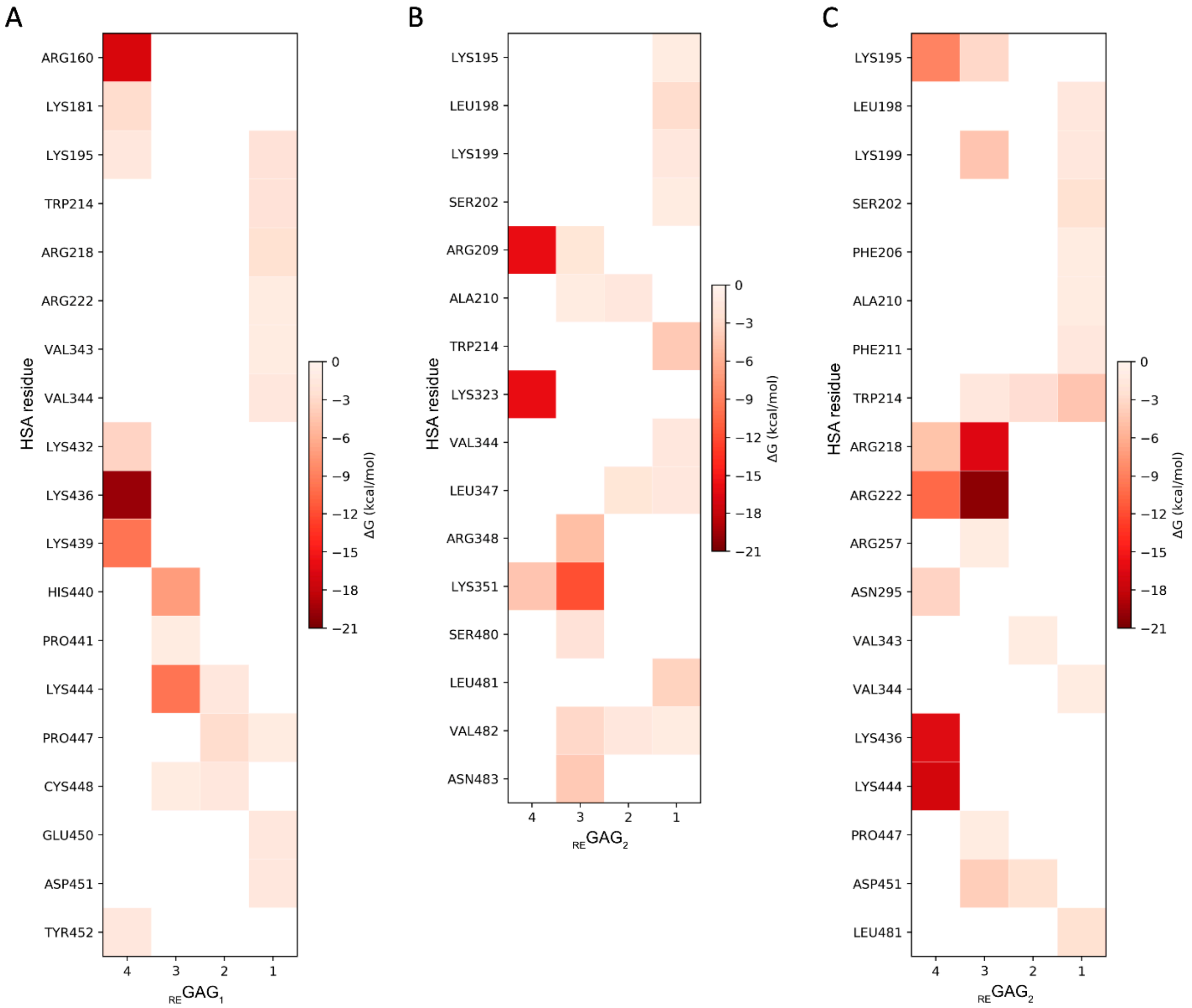

Figure S10. Pairwise binding free energy contributions calculated with MM-GBSA from three independent 100 ns MD simulations of HSA (PDB ID 1H9Z) in complex with (**A**) REGAG1 and (**B**)-(**C**) REGAG2 bound to the FA8 site. MD-refined complexes from Glide ((**A**) and (**C**)) and GlycoTorch (**B**) docking results. Mean values are indicated by the gradient-colored side bar. The different fragments of each REGAG molecule are represented in the x axis by the numbers: (1) biphenyl group, (2) linker, (3) fully sulfated N-acetylglucosamine (GlcNAc), and (4) fully sulfated glucuronic acid (GlcA).

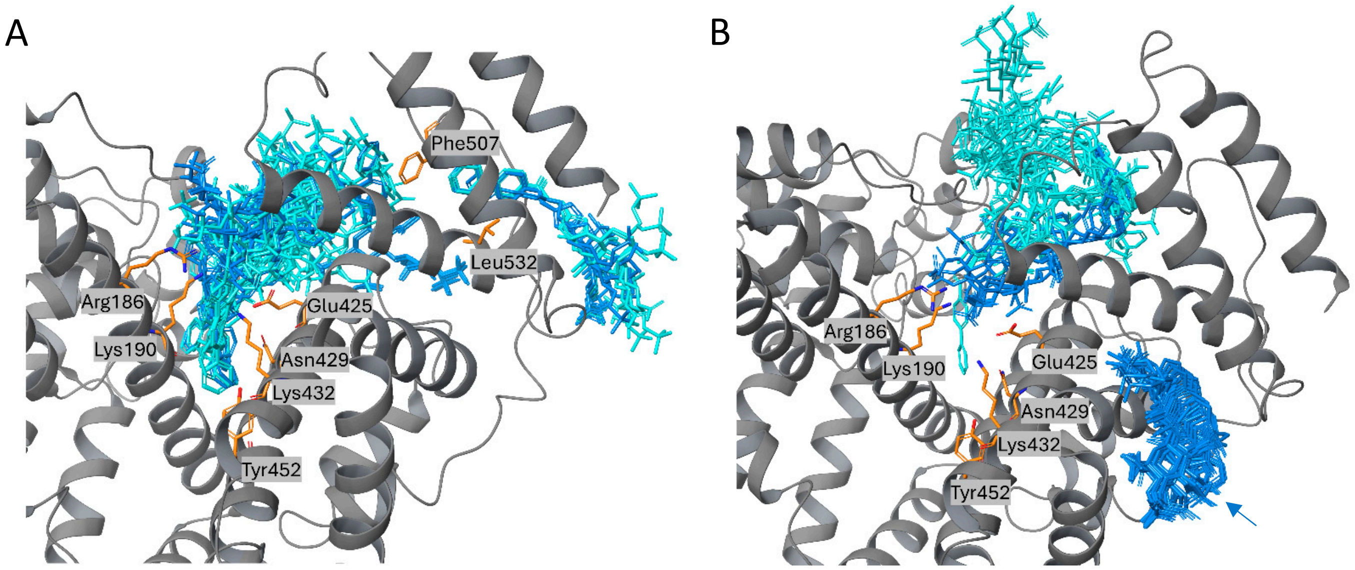

Figure S11. Molecular recognition of the reGAG molecules by HSA. Docking results of reGAG$_1$ (cyan sticks) and reGAG$_2$ (azure sticks) with HSA (PDB ID 1E7A, grey cartoon) using (**A**) GlycoTorch and (**B**) Glide. This view of HSA corresponds to the back side of that shown in the previous figures. Residues reported being involved in recognition in FA9 and FA5 sites are shown in orange sticks and labelled. A cluster uniquely predicted by Glide (B) for reGAG$_2$ at the surface of subdomain IIIB of HSA (marked by an arrow) did not correspond to any known HSA binding site and was therefore excluded from further MD analysis. Figure generated with Maestro v14.3.

pharmaceutics

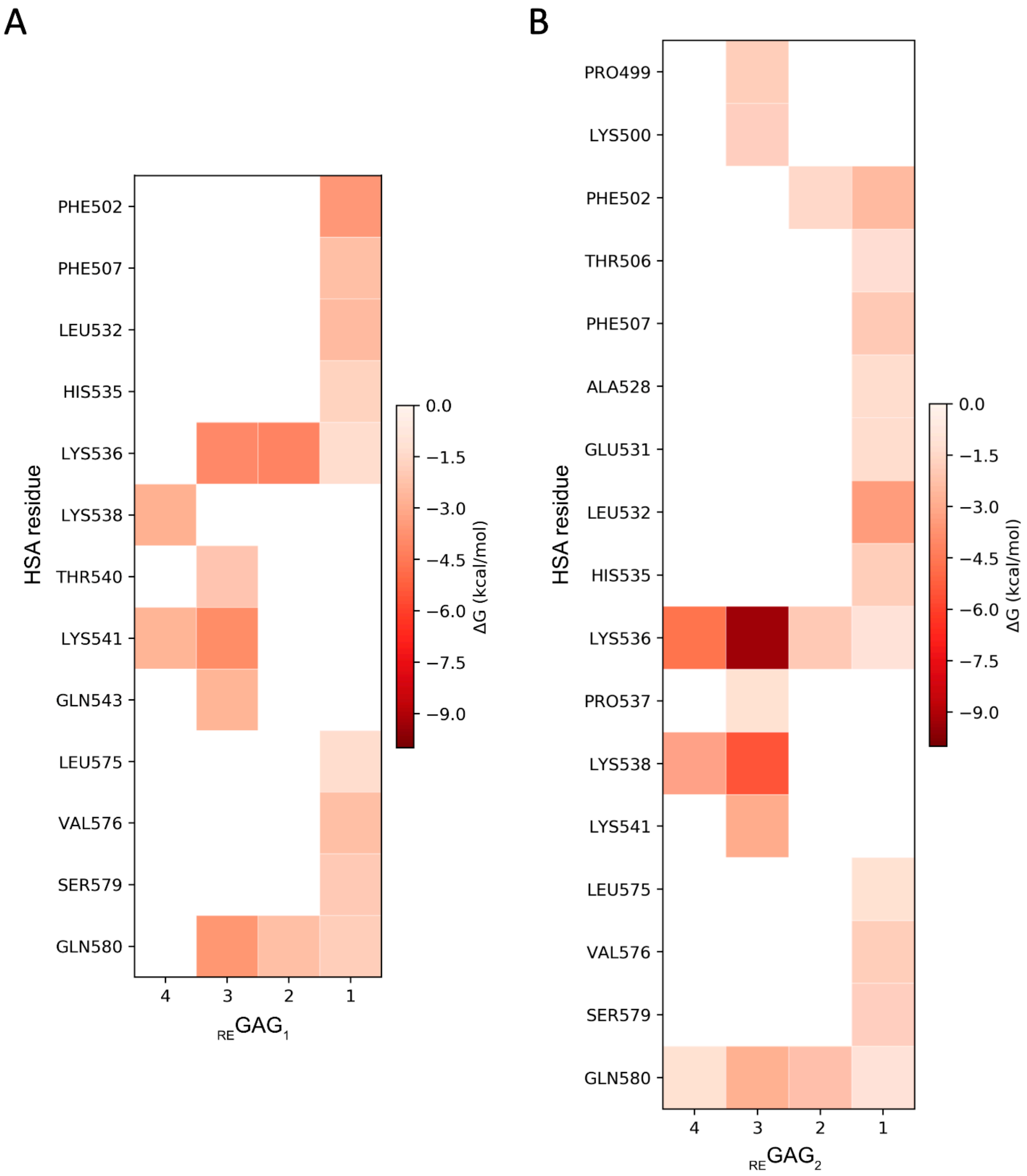

Figure S12. Pairwise binding free energy contributions calculated with MM-GBSA from three independent 100 ns MD simulations of HSA (PDB ID 1E7A) in complex with (**A**) REGAG1 and (**B**) REGAG2 bound to the FA5 site. Mean values are indicated by the gradient-colored side bar. The different fragments of each REGAG molecule are represented in the *x* axis by the numbers: (1) biphenyl group, (2) linker, (3) fully sulfated N-acetylglucosamine (GlcNAc), and (4) fully sulfated glucuronic acid (GlcA).

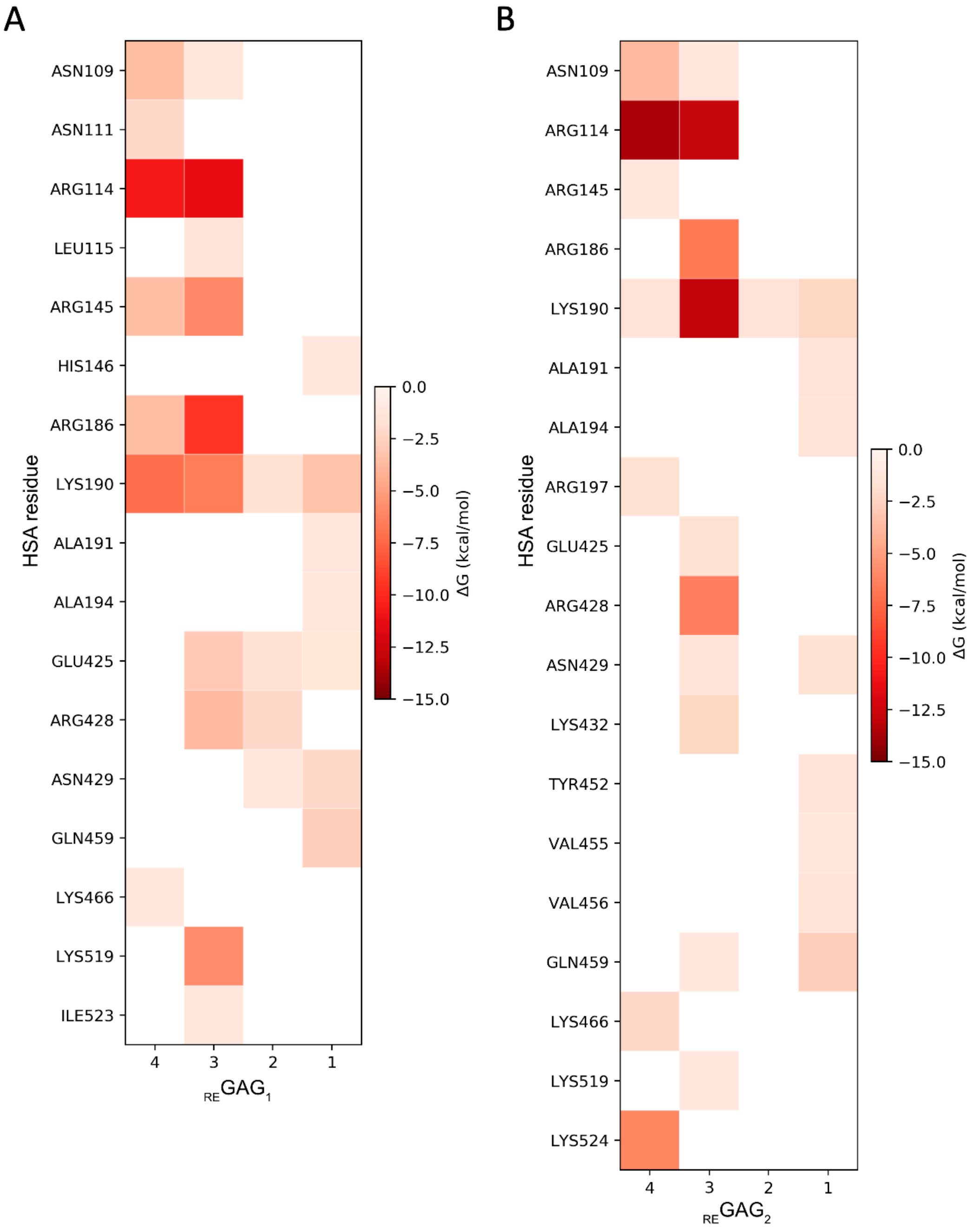

Figure S13. Pairwise binding free energy contributions calculated with MM-GBSA from three independent 100 ns MD simulations of HSA (PDB ID 1E7A) in complex with (**A**) REGAG1 and (**B**) REGAG2 bound to the FA9 site. The results shown correspond to complexes in which the biphenyl groups of REGAG molecules are oriented towards the subdomain IIIA. Mean values are indicated by the gradient-colored side bar. The different fragments of each REGAG molecule are represented in the *x* axis by the numbers: (1) biphenyl group, (2) linker, (3) fully sulfated N-acetylglucosamine (GlcNAc), and (4) fully sulfated glucuronic acid (GlcA).

pharmaceutics MDPI

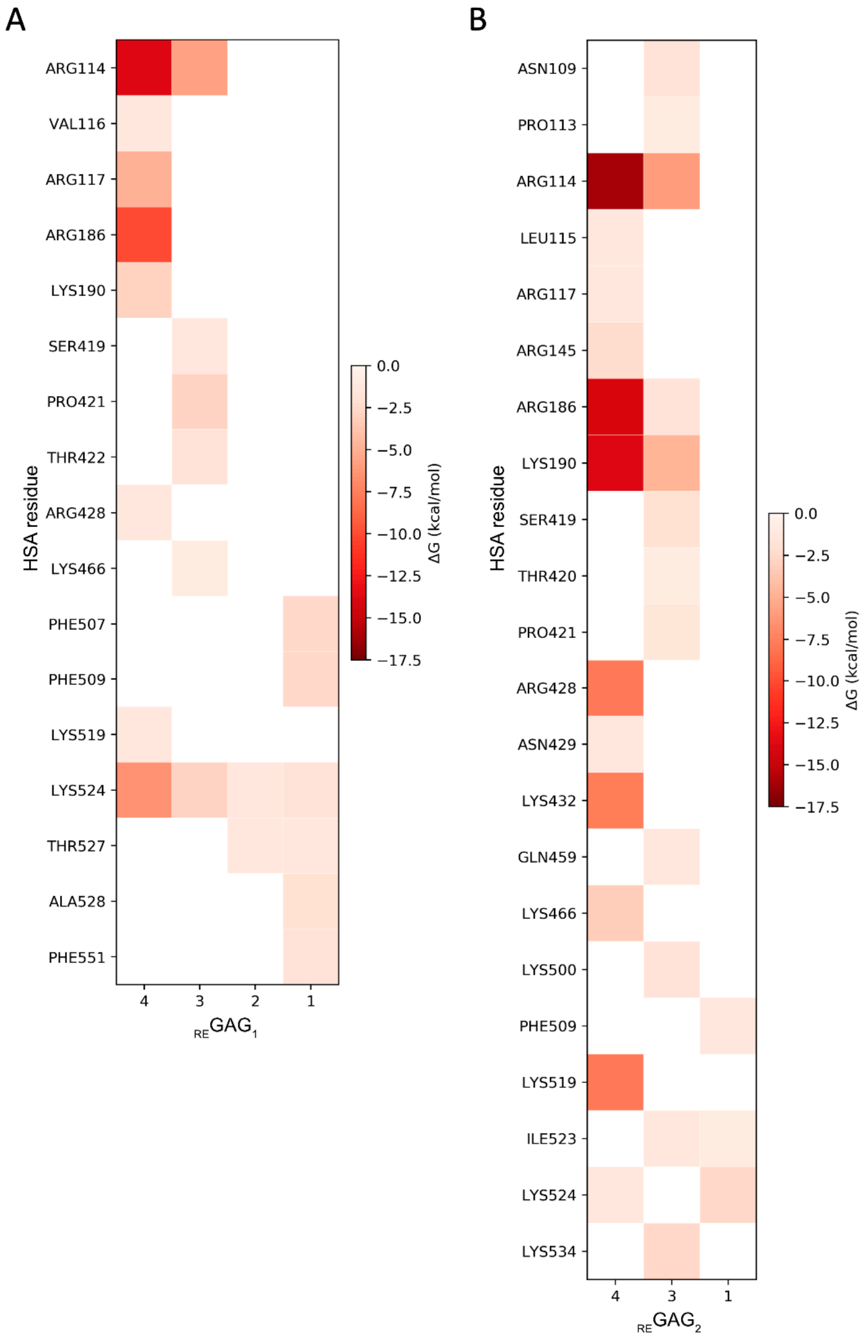

Figure S14. Pairwise binding free energy contributions calculated with MM-GBSA from three independent 100 ns MD simulations of HSA (PDB ID 1E7A) in complex with (**A**) REGAG1 and (**B**) REGAG2 bound to the FA9 site. The results shown correspond to complexes in which the biphenyl groups of REGAG molecules are oriented towards the subdomain IIIB. Mean values are indicated by the gradient-colored side bar. The different fragments of each REGAG molecule are represented in the *x* axis by the numbers: (1) biphenyl group, (2) linker, (3) fully sulfated N-acetylglucosamine (GlcNAc), and (4) fully sulfated glucuronic acid (GlcA).

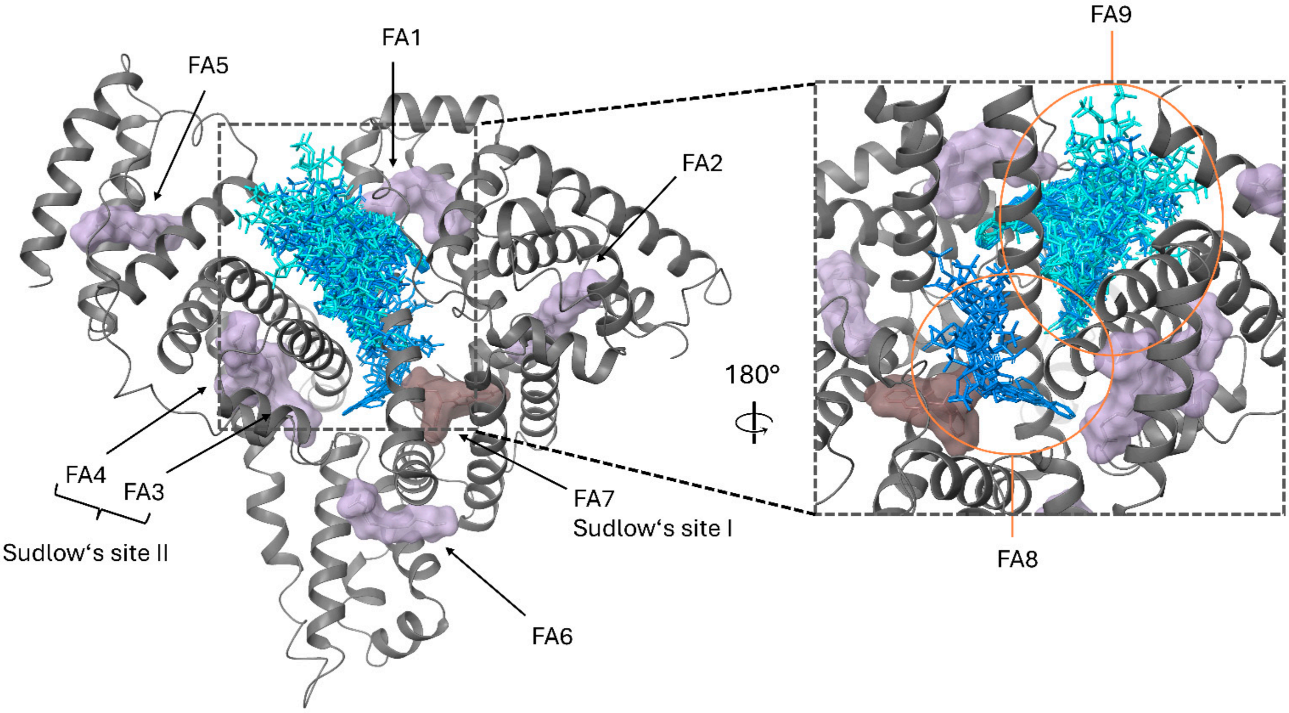

Figure S15. Molecular recognition of REGAG molecules by HSA. Docking results of REGAG1 (cyan sticks) and REGAG2 (azure sticks) in warfarin- and FA-bound HSA (PDB ID 1H9Z, grey cartoon) using GlycoTorch. Bound warfarin and FA are highlighted in brown and purple transparent surfaces, respectively. Binding poses at the FA8 and F9 sites are zoomed in and indicated with orange circles. Figure generated with Maestro v14.3.

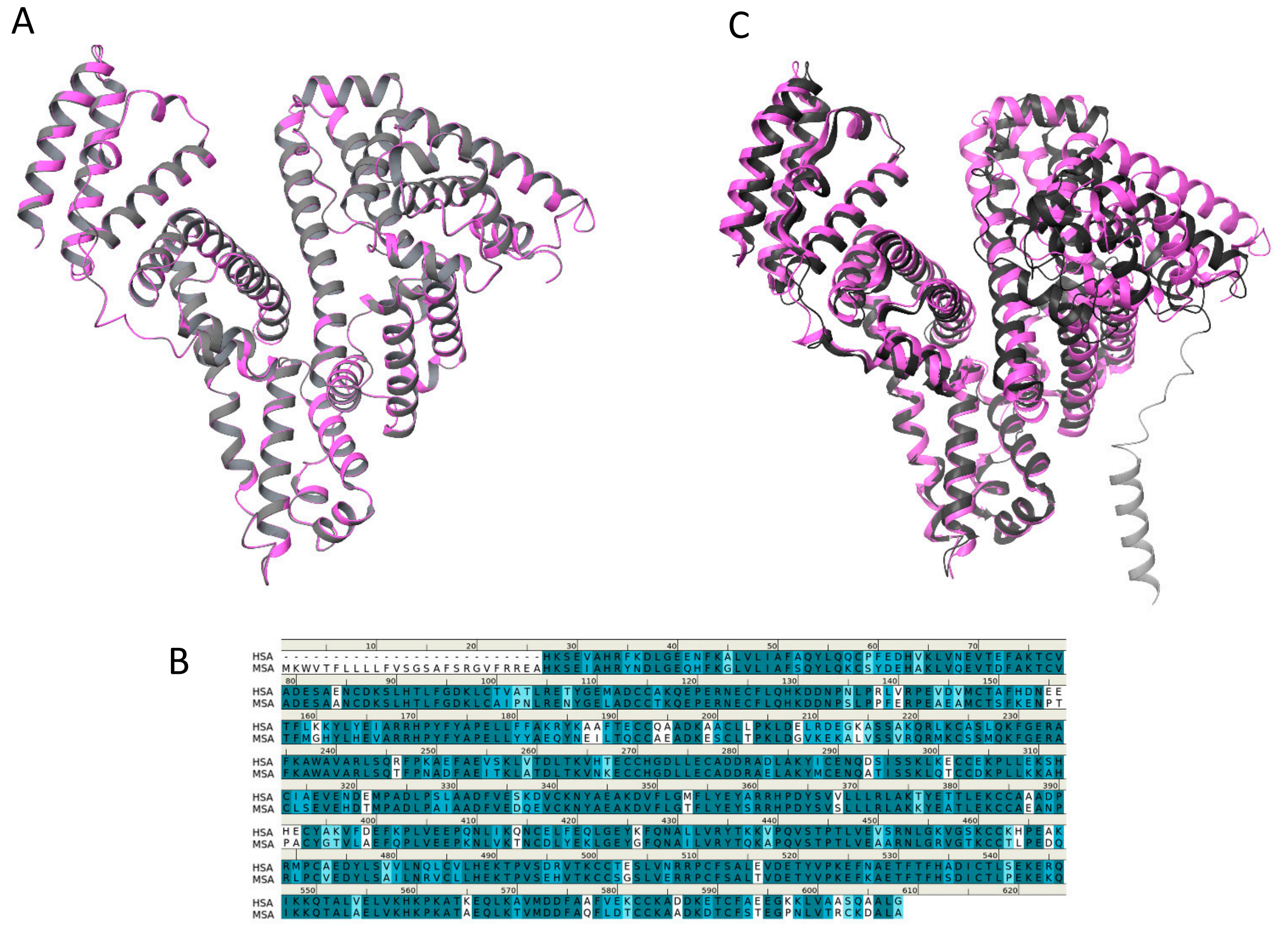

Figure S16. Molecular modeling of MSA. (**A**) Superimposition of the 3D molecular model of MSA (magenta cartoon) obtained by comparative modeling with the crystallographic structure of HSA (PDB ID 8RCO, grey cartoon) as template. (**B**) Sequence alignment of HSA and MSA used for comparative modeling. Conserved, semi-conserved and non-conserved residues are highlighted with teal, blue and white background, respectively. (**C**) Superimposition of the MSA 3D model with the available model by AlphaFold (AF-P07724-F1, black cartoon). The AlphaFold model includes a low-confidence N-terminal region (residues Met1-Ala26, pLDDT < 50, light grey cartoon). Figure generated with Maestro v14.3 (A, C) and BIOVIA (B).

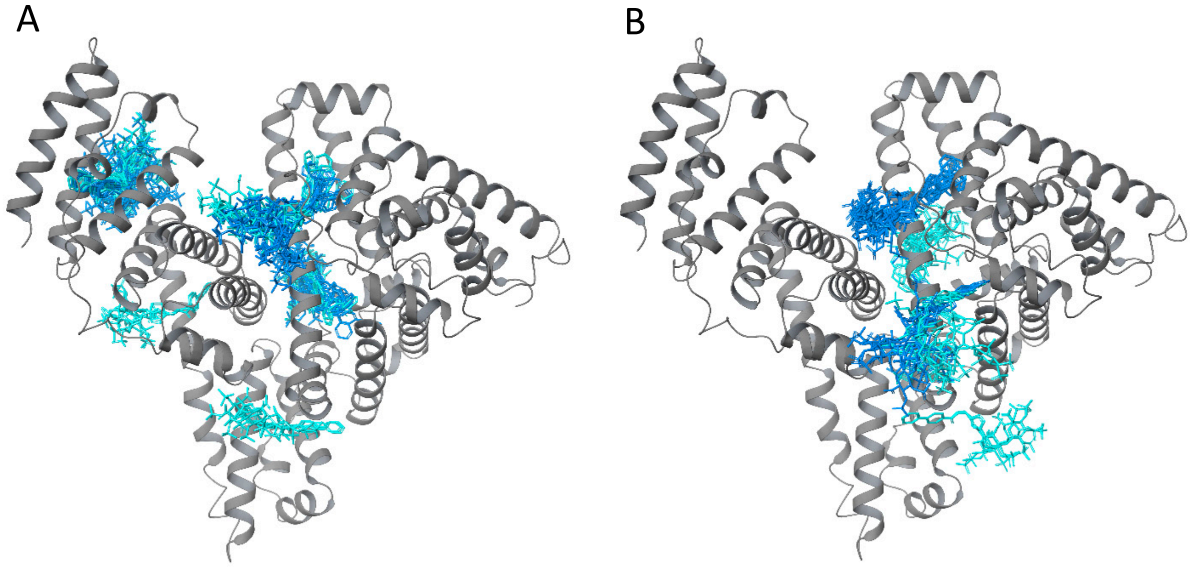

Figure S17. Molecular recognition of REGAG molecules by MSA. Docking results of REGAG$_1$ (cyan sticks) and REGAG$_2$ (azure sticks) with the MSA model using (**A**) GlycoTorch and (**B**) Glide. Figure generated with Maestro v14.3.

pharmaceutics

MDPI

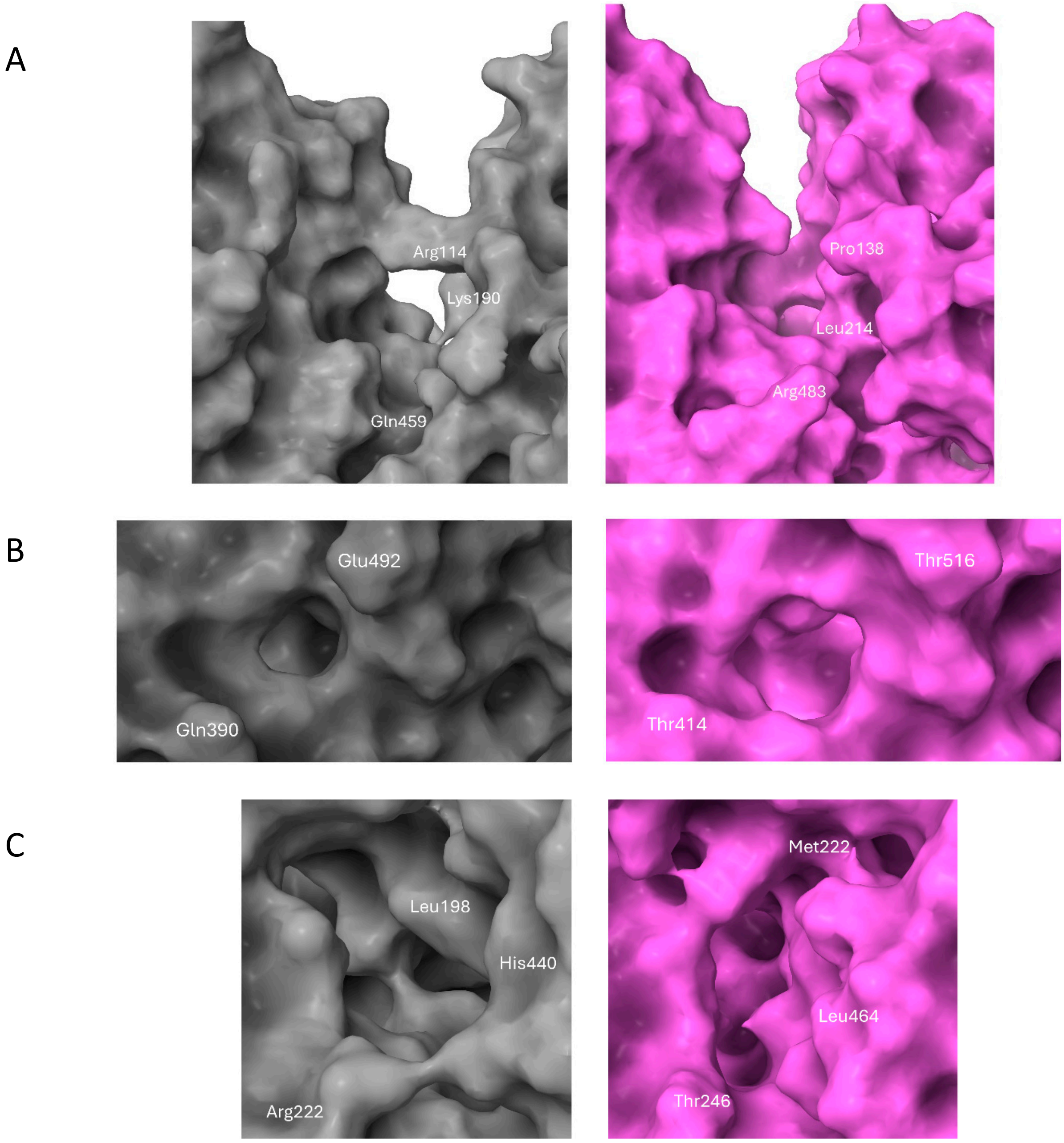

Figure S18. Comparative of HSA (left, in grey) and MSA (right, in magenta) recognition sites (shown as molecular surfaces). Non-conserved residues between the human and murine proteins are labeled. (**A**) FA9 site (HSA PDB ID 1E7A), (**B**) FA3/4 (drug-binding site II, PDB ID 1E7A), (**C**) FA8 site (HSA PDB ID 1H9Z). Figure generated with Maestro v14.3.

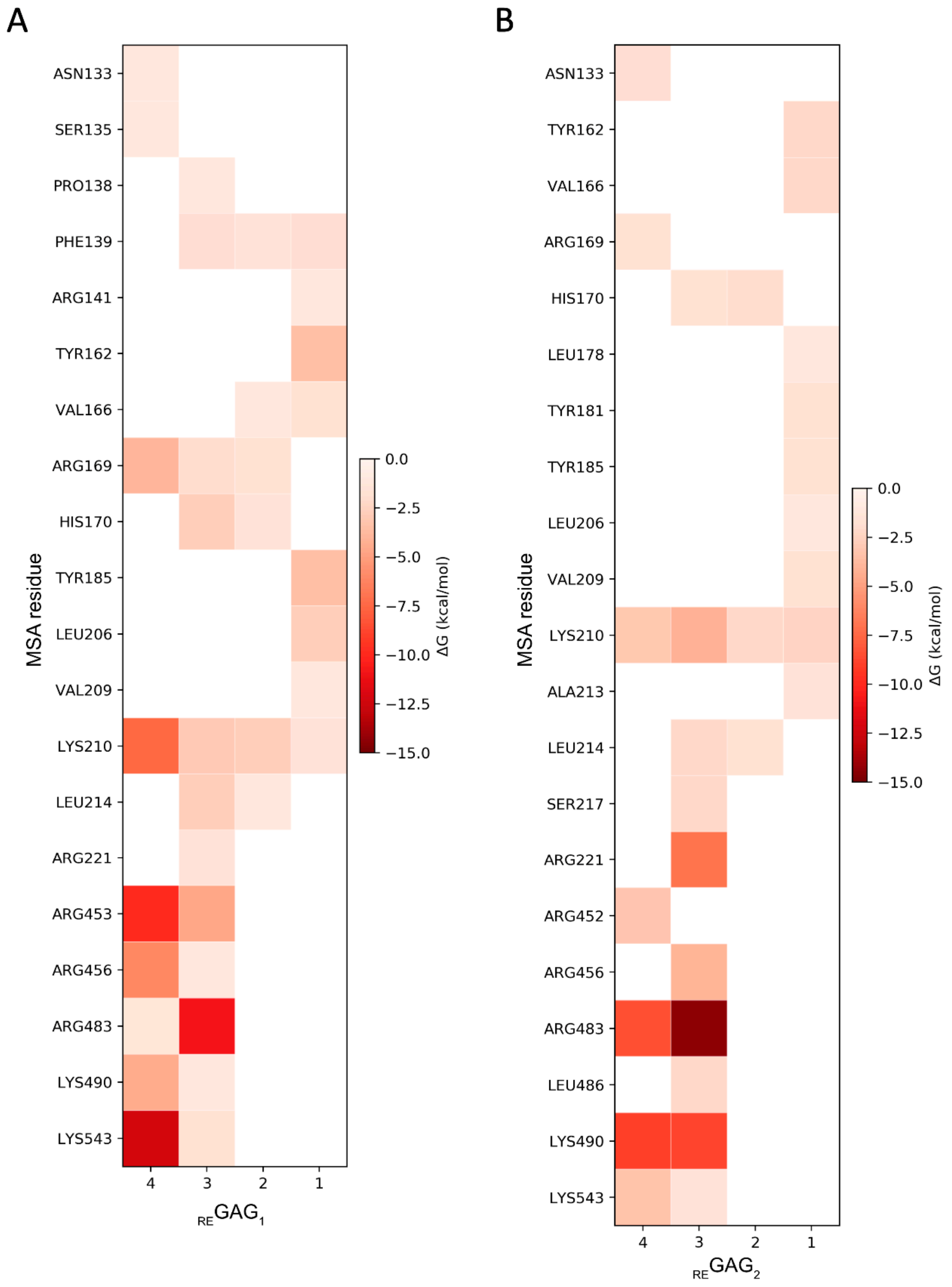

Figure S19. Pairwise binding free energy contributions calculated with MM-GBSA from three independent 100 ns MD simulations of MSA in complex with (**A**) REGAG1 and (**B**) REGAG2 bound to the FA1 site. Mean values are indicated by the gradient-colored side bar. The different fragments of each REGAG molecule are represented in the *x* axis by the numbers: (1) biphenyl group, (2) linker, (3) fully sulfated N-acetylglucosamine (GlcNAc), and (4) fully sulfated glucuronic acid (GlcA).

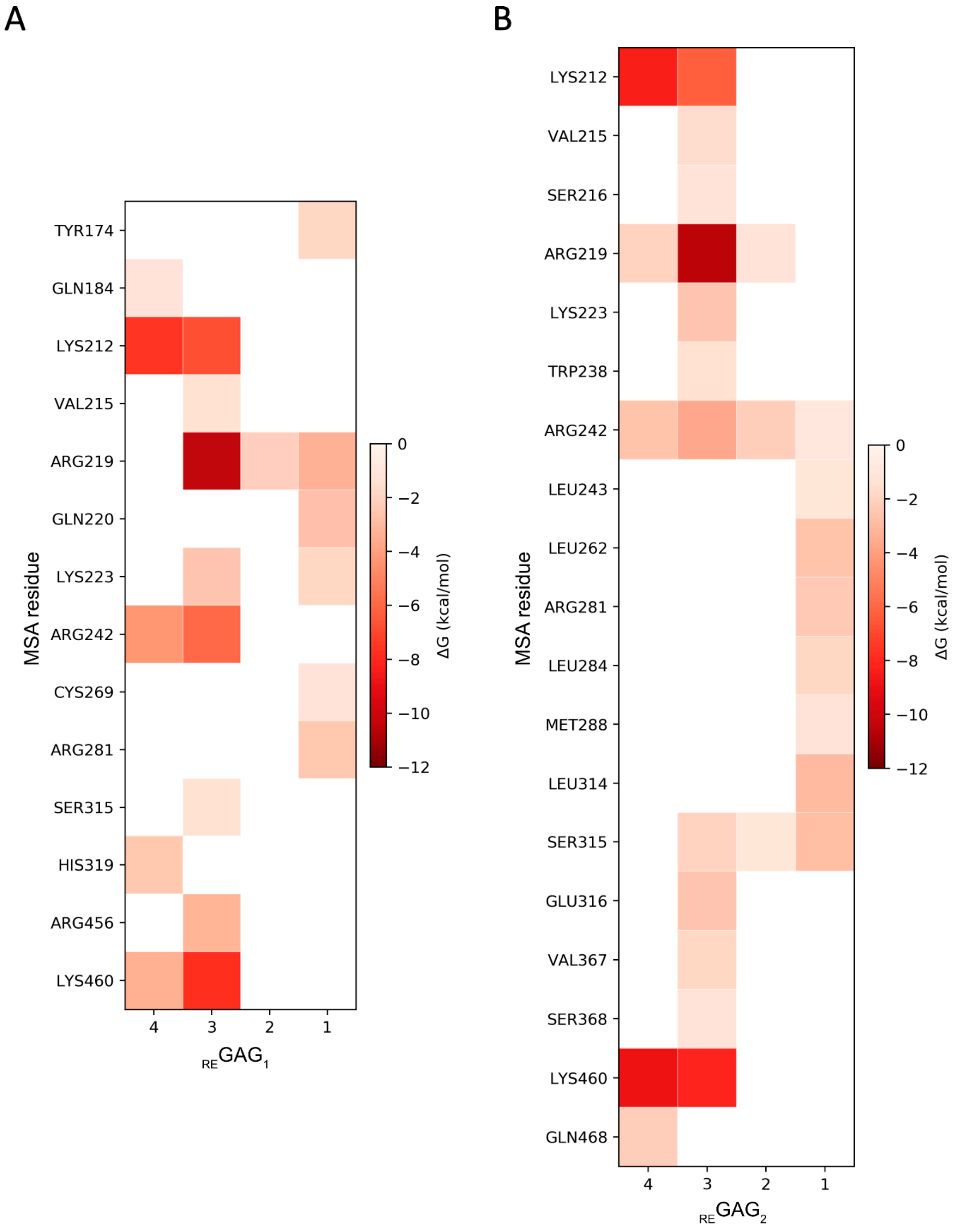

Figure S20. Pairwise binding free energy contributions calculated with MM-GBSA from three independent 100 ns MD simulations of MSA in complex with (**A**) REGAG1 and (**B**) REGAG2 bound to the FA7 site. Mean values are indicated by the gradient-colored side bar. The different fragments of each REGAG molecule are represented in the *x* axis by the numbers: (1) biphenyl group, (2) linker, (3) fully sulfated N-acetylglucosamine (GlcNAc), and (4) fully sulfated glucuronic acid (GlcA).

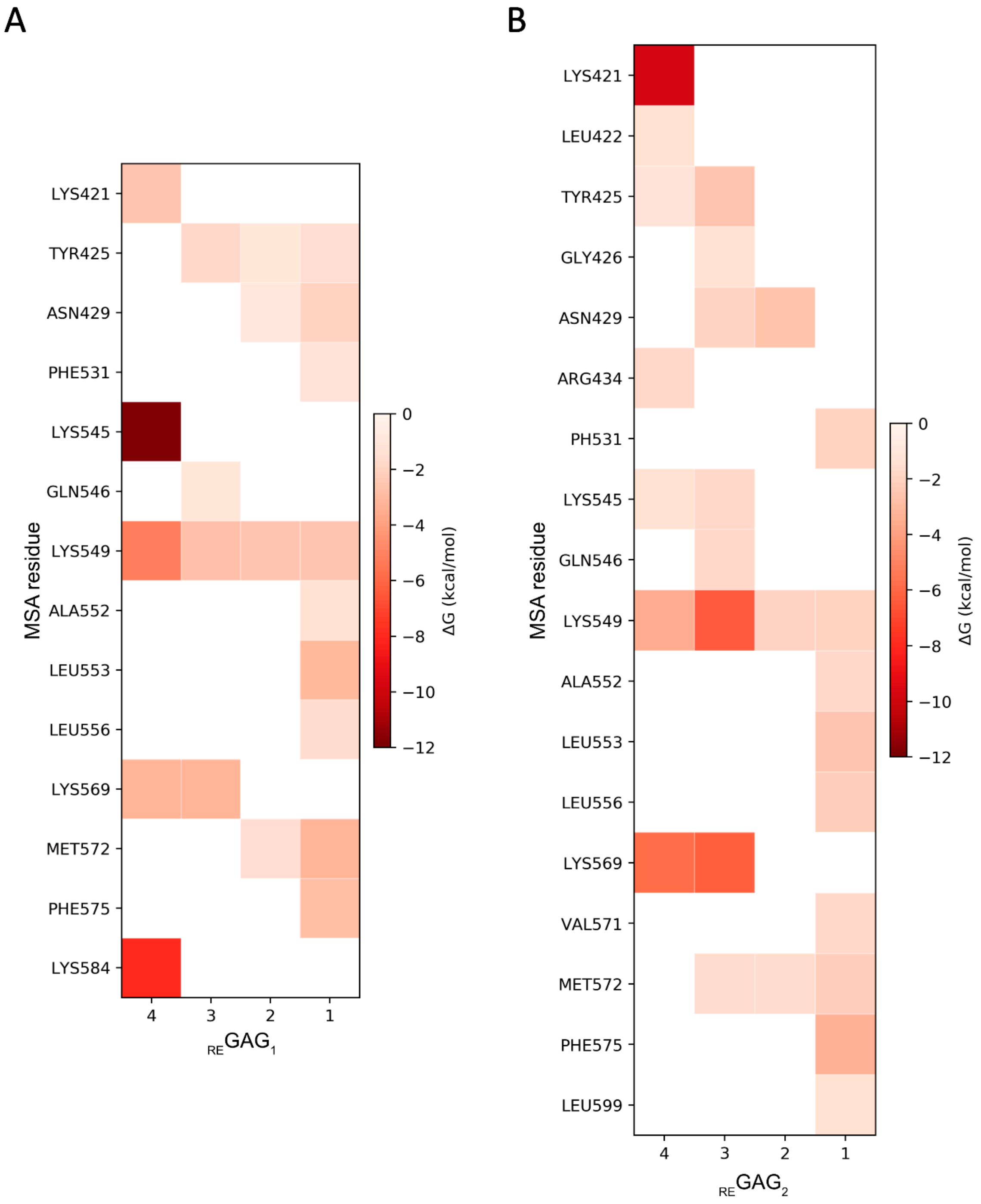

Figure S21. Pairwise binding free energy contributions calculated with MM-GBSA from three independent 100 ns MD simulations of MSA in complex with (**A**) REGAG1 and (**B**) REGAG2 bound to the FA5 site. Mean values are indicated by the gradient-colored side bar. The different fragments of each REGAG molecule are represented in the *x* axis by the numbers: (1) biphenyl group, (2) linker, (3) fully sulfated N-acetylglucosamine (GlcNAc), and (4) fully sulfated glucuronic acid (GlcA).
